# Biotechnological Valorization of Food Marine Wastes: Microbial Productions on Peptones Obtained from Aquaculture By-Products

**DOI:** 10.3390/biom10081184

**Published:** 2020-08-14

**Authors:** José Antonio Vázquez, Ana I. Durán, Araceli Menduíña, Margarita Nogueira

**Affiliations:** Grupo de Reciclado y Valorización de Materiales Residuales (REVAL), Instituto de Investigaciones Marinas (IIM-CSIC), C/Eduardo Cabello, 6, CP 36208 Vigo, Galicia, Spain; anais@iim.csic.es (A.I.D.); araceli@iim.csic.es (A.M.); marga@iim.csic.es (M.N.)

**Keywords:** aquaculture by-products, marine waste valorization, marine peptones, bacterial bioconversion of marine waste, marine waste circular bioeconomy

## Abstract

Based on a biotechnological strategy, in the present work several peptones are produced from the Alcalase hydrolysis (0.1–0.2% *v/w*, 56–64 °C, pH 8.27–8.98, 3 h) and thermal processing (105 °C, 60 min) of wastes generated from the industrial processing of turbot, salmon, trout, seabream and seabass. These peptones were included (in the range of 2.6–11 g/L of soluble protein) as main source of organic nitrogen (protein substrates) in low-cost media for the culture of lactic acid bacteria (LAB), marine probiotic bacteria (MPB) and ubiquitous Gram+ bacteria. In most cases, batch fermentations conducted in aquaculture peptone media led to the best growth, metabolic productions and yields. Nevertheless, no significant differences between aquaculture peptones and commercial media were generally observed. Kinetic parameters from a logistic equation and used for cultures modeling were applied with the purpose of comparing the bioproduction outcomes. In economical terms, the validity of the aquaculture peptones as substitutives of the peptones (meat extract, casitone, etc.) from commercial media was also compared. The decreasing of the costs for LAB bioproductions ranged between 3–4 times and the growth costs of MPB and Gram+ bacteria were improved more than 70 and 15 times, respectively, in relation to those found in control commercial media.

## 1. Introduction

World aquaculture production, including aquatic plants, achieved 112 MT in 2017, exceeding 18 MT more than inland captured and marine fish [1,2]. Due to the overexploitation of wild fishing stocks and the increasing presence of heavy metals on many fish species, the demand for aquaculture products is growing and production could reach 60% of all aquatic food in the coming years [1]. About 80 MT are from food fish farming as finfish (54 MT), molluscs (17 MT), crustaceans (8 MT) and other aquatic animals (1 MT), considering that China is the largest producer with almost 62% of total aquaculture fish. In Europe, more than 2 MT were produced in 2017, maintaining a constant production in the last quinquennium. Excluding molluscs, the most important fish farming species in Europe are salmon, trout, seabream, seabass and turbot [1,2]. In the south of Europe, aquaculture fish are mainly marketed as whole individuals in comparison with the rest of the continent where fillet presentations are, in some cases, the only way of commercialization of fish in the hypermarkets. Unfortunately, the globalization and our growing consumption habits are dramatically increasing the sale of clean fillets packaged in plastic trays. The operations applied to the aquaculture species for this food processing (headed, gutted and filleted) generates a huge volume of wastes (head, viscera, trimmings and frames) that must be managed and, mainly, valorized to avoid pollution problems, to improve the sustainability of the aquaculture sector and to obtain bioproducts of high-added value [3,4,5]. The total percentage of these residues in relation to the initial individuals depends on the species and ranges from 35–45% in the case of salmonids to 60% for turbot and seabream [5,6].

The most common way of managing such wastes is by means of the production of fishmeal and oils [7,8,9]. However, the composition of the fish wastes is rich in protein, oil and mineral materials that can be adequate for the application of a more specific process for its recovery [9,10,11]. One of the most interesting protocols of valorization, in frame with the objectives of the circular economy, is using enzyme proteolysis, in optimal conditions, for the digestion of these substrates and the production of oils, bioactives and fish protein hydrolysates [12,13,14] of potential interest in the formulation of nutraceutical, animal feed and human nutrition supplements [15,16,17]. Nevertheless, the number of articles is surprisingly limited. In this context, other potential high-added bioproducts are the peptones; that is, a whole of proteins, peptides and free amino acids useful as ingredients (source of organic nitrogen) in the complex media needed for the growth of microorganisms [18].

Peptones are commonly the most expensive component (ranging from 80–300 €/kg) in the culture broths and their name and composition is varied and dependent on the origin of the materials and the type of processing applied for their production: casitone (from casein), meat extract (from bovine meat), soy peptone (from soy) or tryptone (casein hydrolyzed by trypsin), etc. [19]. Although the peptones from fish are scarcely marketed, several marine by-products (heads, viscera, effluents from tuna, squid, cod, etc.) have successfully supported the growth of various bacteria and the concomitant production of interesting metabolites [20,21,22,23,24]. However, the recovery of peptones from residues of aquaculture and its uses in bacterial productions is almost completely unexplored. To our knowledge, only the production of two bacteriocins (nisin and pediocin) from *Lactococcus lactis* and *Pediococcus acidilactici*, respectively, have been studied in a medium including a peptone obtained by the autohydrolysis of trout viscera [25].

In the present work, several peptones obtained by enzymatic hydrolysis or by thermal processing of aquaculture by-products (head, viscera, trimmings + frames) have been studied as protein ingredients on alternative media for the growth of three kinds of bacteria: lactic acid bacteria (LAB), marine probiotic bacteria (MPB) and ubiquitous Gram+ bacteria. In all cases, the capacity of these low-cost media to support bacterial growth was excellent and the kinetics of fermentations and metabolite productions were accurately simulated by the logistic equation. Additionally, the economical evaluation of the bioproductions generated in the aquaculture peptones, in relation to the results in the control commercial media demonstrate the validity of this biotechnological approach to valorize fish farming wastes. This is the first manuscript dealing with the production and application of peptones from aquaculture wastes to the growth of several bacterial strains of technological interest.

## 2. Materials and Methods

### 2.1. Wastes from Aquaculture Food Processing

**Figure 1:**
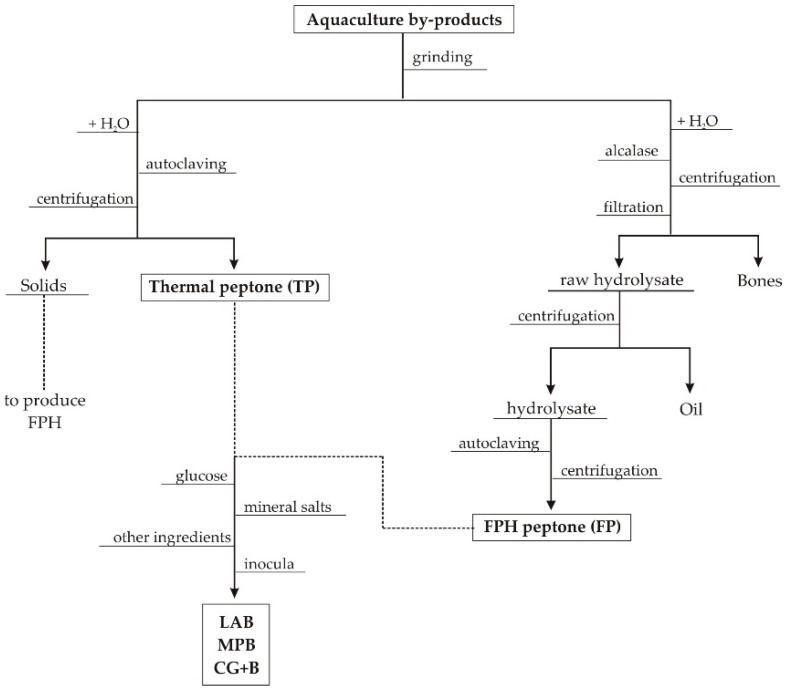
Schematic flowchart of aquaculture wastes to produce peptones for bacterial fermentations.

Aquaculture wastes produced in the food processing (mainly filleting) of salmon (*Salmon salar*, Sa), rainbow trout (*Oncorhynchus mykiss*, RT), turbot (*Scophthalmus maximus*, Tu), gilt-head seabream (*Sparus aurata*, Sb) and European seabass (*Dicentrarchus labrax*, Sbass) were kindly supplied by Isidro 1952, S.L. (Cambre, A Coruña, Spain), Dr. Johan Johansen (Salten Havbrukspark, Nygårdsjøen, Norway), Prodemar (Stolt Sea Farm S.A., Carnota, A Coruña, Spain) or purchased from a local market. These materials were stored at –18 °C until peptones production. The three types of residues were heads (He), trimmings and frames (Tu) and viscera (Vis). Initially, materials were ground in a meat mincer and separated into two portions to prepare the thermal and hydrolyzed peptones (Figure 1). In the first case, substrates were mixed with distilled water (ratio S:L = 1:1) and autoclaving at 105 °C for 60 min. Then, solutions were centrifuged (15000× *g*/20 min) and liquid fraction was called thermal peptone (TP).

The other aliquot of grinded wastes were hydrolyzed in a controlled pH-Stat system with a 5 L glass-reactor (5M NaOH as alkaline reagent). Thus, distilled water was added to each substrate in a (S:L) ratio of 1:1 and Alcalase 2.4 L was used at a concentration of 0.1% (*v*/*w*) for RT and 0.2% ((*v*/*w*) for Sa, Tu, Sb and Sbass [6,26]. Hydrolysis were performed at 56.2 °C and pH 8.27 (Sa), 64.2 °C and pH 8.98 (RT), 60.3 °C and pH 8.82 (Tu), 56 °C and pH 8.30 (Sb) and 56 °C and pH 8.30 (Sbass). All hydrolysis were run for 3 h and under continuous agitation at 200 rpm. At the end of the hydrolysis, the content of the reactors was filtered (100 μm) to remove bones, the liquid hydrolysates were centrifuged (15,000× *g*/20 min) to separate oils and liquid supernatant were subsequently autoclaved (105 °C/20 min) and finally centrifuged (15,000× *g*/20 min) to obtain the FPH peptone (FP).

### 2.2. Microbiological Methods and Culture Media

Six bacteria from different genus and characteristics were assayed: (a) two lactic acid bacteria (LAB) from CECT (Spanish Type Culture Collection), *Lactobacillus plantarum* CECT 220 and *Lactobacillus brevis* CECT 4043; (b) two marine probiotic Gram− bacteria (MPB), kindly provided by Dr. Lone Gram (DTU Aqua, Denmark), *Phaeobacter* sp. DIFR 27-4 and *Pseudomonas fluorescens* DIFR AH-2; and (c) two common Gram+ bacteria (CG+B) from CECT, *Bacillus subtilis* CECT 35 and *Staphylococcus epidermidis* CECT 231. The commercial media employed for conservation, inocula and control purposes were: Man, Rogosa and Sharpe medium (MRS, from Pronadisa, Hispanlab S.A., Spain) for LAB, marine medium (MM, Difco, Becton, Dickinson and Company, MD, USA) for MPB and tryptone soy broth (TSB, Panreac Química, Spain) for CG+B. Stock cultures were always preserved at −80 °C in each specified media including 25% glycerol (*w/w*). The concentration of soluble protein in the alternative media (with aquaculture peptones) was established by substituting the Lowry protein content in commercial MRS (10 g/L from meat extract and bactopeptone), commercial MM (2.6 g/L from commercial peptone) and TSB (11 g/L from casitone and soy peptone) (Table S1). Fermentations were carried out in duplicate using 300 mL Erlenmeyer flasks with 180 mL of medium at 30 °C (LAB and *B. subtilis*), 22 °C (MPB) and 37 °C (*S. epidermidis*) and 200 rpm of orbital shaking. In all cases, the initial pHs were fixed to 6.0, 7.5 and 7.0 with NaOH 5 N and culture broths were finally sterilized separately at 121 °C for 15 min. Inocula (0.5% *w/v*) consisted of cellular suspensions from 12–16 h cultures on control medium.

### 2.3. Sampling and Determinations of Growth and Metabolites

Samples from each culture were collected at pre-determined times and divided into two aliquots. The first one was employed in the quantification of the viable cells using plate count in MRS agar medium for LAB, MM agar medium for MPB and TSB for CG+B. Serial tenfold dilutions were prepared in peptone-buffered solutions, and 0.1 mL samples were extended in plate by triplicate, incubated at 30 °C for 48 h, and manually counted. The obtaining results were expressed as G = ln(N/N_0_), where N is the colony-forming units per mL (cfu/mL) and N_0_ is the initial colony-forming units per mL (cfu/mL). The second aliquot was centrifuged at 3273× *g* for 15 min, from which the supernatant was used for determining the content of soluble proteins, reducing sugars and lactic and acetic acids. The precipitate was washed and resuspended in distilled water at an appropriate dilution to measure the optical density (OD) at 700 nm and then dry weight was estimated from a calibration curve (OD vs. dry weight).

Aquaculture peptones and fermented media free of bacteria were analyzed, in duplicate, as follows: (1) Reducing sugars (RS) were quantified by means of 3,5-dinitrosalicylic reaction [27]; (2) total soluble proteins (Pr) were obtained using the method of Lowry [28]; (3) total nitrogen by Havilah method [29], applied to digests obtained through the classic Kjeldahl procedure, (4) total sugars (TS) content was determined by the protocol of Dubois et al. [30]; (5) amino acid presence was measured following the method of Moore et al. [31], employing an Amino Acid Analyser (Biochrom 30 series, Biochrom Ltd., Cambridge, UK). Additionally, the organic acid metabolites (lactic and acetic acids) were quantified by HPLC, after filtration of the samples (0.22 μm Millex-GV, Millipore, Burlington, MA, USA), using an ION-300 column (Transgenomic, Omaha, NE, USA) with 6 mM sulphuric acid as mobile phase (flow = 0.4 mL/min) at 65 °C and a refractive-index detector [32].

### 2.4. Mathematical Modelling of Bacterial Kinetics

The experimental growth, bacterial biomass as dry weight (*X*) and cell formation (*G*), together with lactic acid production (*L*) and acetic acid production (*A*) in LAB, were modelled by the following logistic equation [33]:

(1) $$P=\frac{P_{m}}{1+\exp\left[2+\frac{4v_{P}}{P_{m}}\left(\lambda_{P}-t\right)\right]}$$

Other parameters from Equation (1) were additionally determined in order to know the rest of sigmoid experimental profile [33]:

(2) $$\mu_{P}=\frac{4v_{P}}{P_{m}}$$

(3) $$\tau_{P}=\lambda_{P}+\frac{2}{\mu_{P}}$$

(4) $$t_{mP}=\tau_{P}+\frac{P_{m}}{2v_{P}}$$

where *P* is the bioproduct determined (*X*, *G*, *L* or *A*); *t* is the time of culture (h), *P_m_* is the maximum product production (g/L for *X*, *L* and *A* and dimensionless for *G*); *v_P_* is the maximum production rate (g L^−1^, h^−1^ for *X*, *L* and *A* and h^−1^ for *G*); *λ_P_* is the products lag phase (h); *μ_P_* is the specific maximum production rate (h^−1^); *τ_P_* is the time required to achieve the half of the maximum production (h) and *τ_mP_* is the time required to reach the maximum production (h). Moreover, the yields of bioproductions on soluble protein uptake (*Y_P_*/*Y_Pr_*) and reducing sugars (*Y_P_*/*Y_RS_*) consumption were determined.

### 2.5. Economical Evaluation of Aquaculture Peptones for Bioproductions

A simple and initial study of economical sustainability of the bacterial production costs was performed. Based on the market prices of MRS, MM and TSB ingredients, as well as the values of *X_m_*, *G_m_*, *L_m_* and *A_m_* summarized in Tables S3 and S14, the cost of production of biomass (in €/g), cells (in €/cell), lactic (in €/g) and acetic (in €/g) acids were estimated for each residual and control media. The reference prices of peptones employed were: beef extract (200 €/kg), bactopeptone (152 €/kg), peptone (90 €/kg), casitone (200 €/kg) and soypeptone (90 €/kg). In these calculations the costs of production of aquaculture peptones (energy and reagents demand) have not been incorporated. These costs are highly dependent on the production scale of peptones, they are proportionally higher at lab scale than at industrial size and difficult to quantify on a laboratory scale. Even so, these peptone processing costs should not suppose an increase of more than 30% in the production cost in the least conservative option [34].

### 2.6. Numerical Fittings and Statistical Analyses

Fitting procedures and parametric estimations calculated from the results were carried out by minimising the sum of quadratic differences between the observed and model-predicted values, using the non-linear least-squares (quasi-Newton) method provided by the macro-“Solver” of the Microsoft Excel spreadsheet. Confidence intervals from the parametric estimates (Student’s t test) and consistence of mathematical models (Fisher’s F test) were evaluated by “SolverAid” macro (Levie’s Excellaneous website: http://www.bowdoin.edu/~rdelevie/excellaneous).

## 3. Results and Discussion

### 3.1. Production and Characteristics of Aquaculture Peptones

All the wastes generated in the industrial processing of the most important aquaculture species in Europe (heads, viscera, trimmings together with frames of turbot, salmon, trout, seabream and seabass) were studied in the present work as source of peptones (mainly organic nitrogen in form of proteins, peptides and free amino acids) for the culture of several bacteria. Table 1 shows the basic composition of peptones in terms of soluble protein, total nitrogen, reducing and total sugars content. The levels of proteins varied from 33 to 81 g/L for FP and 19 to 73 g/L for TP, reaching higher concentrations in FP than those found in TP. In many cases, the efficiency of protein recovery from thermal peptones processing was less than half of what was obtained, including a previous enzymatic hydrolysis step (FPH peptones). Similar results were observed for the total nitrogen content in peptones. Alcalase was selected as a biocatalyst because it is one of the commercial proteases most widely used in the hydrolysis of fish substrates due to its ease of application, cost-effectiveness, high activity and ability to digest several types of marine by-products [35,36,37,38,39]. The residual material generated from TP production, still rich in proteinaceous substrates, could be further hydrolysis or included as a source of fishmeal. In general, the lowest contents of thermal peptones were extracted from viscera in comparison with the other two substrates.

Moreover, Sb_He, Sbass_TF, Tu_He and Tu_TF showed the highest values of soluble protein recovered and trout materials led to the lowest amount of protein. The content in amino acids was also determined (Table 2 and Table 3), revealing some differences between peptones: the percentage ranges of glycine (7.5–21.4%), proline (4.9–9.6%), OHproline (2.3–7.3%) and alanine (6.7–10.3%) were higher in TP and tyrosine (2.9–4.4%), phenylalanine (4.0–7.2%) and aspartic acid (8.8–10.8%) were larger in FP. The greater content of Gly, Pro and OHPro may indicate the presence of collagen protein derivatives in TP peptones. The level of glutamic acid varied in a quite similar interval in both cases. The average molecular weights of the peptides present in FP, determined by gel permeation chromatography (GPC) [6], were around 700–1500 Da with 5% of peptides larger than 3 kDa. In the case of TP, those values ranged mostly between 50–640 kDa (data not shown) since no specific protein hydrolysis was applied.

The concentration of total sugars in peptones (Table 1) was always lower than 2 g/L and from these, less than half was in the form of reducing sugars, mainly glucose, (<0.5 g/L). Taking into account these concentrations, the presence of these sugars in the culture media was irrelevant (as much 0.73 g/L of total sugars and 0.18 g/L of reducing sugars in the medium formulated with Sb_TF peptone) due to the dilution needed to fix the definitive concentration of 11 g/L or 10 g/L of Lowry-protein in the alternative TSB and MRS media.

### 3.2. LAB Bioproductions on Marine Peptones from Aquaculture Wastes

LAB are microorganisms defined as fastidious because they require complex media formulated with several components as inorganic salts, glucose, tensioactive and different peptones [40,41]. They are of great importance in the industry and, especially, two strains of *L. plantarum* and *L. brevis* have been chosen as target bacteria to test the suitability of aquaculture peptones due to its technological properties as starters of different fermented foods based on dairy, meat and vegetable substrates [42,43,44]. In addition, they also showed probiotic functions in human and aquaculture diseases [45,46,47].

**Figure 2:**
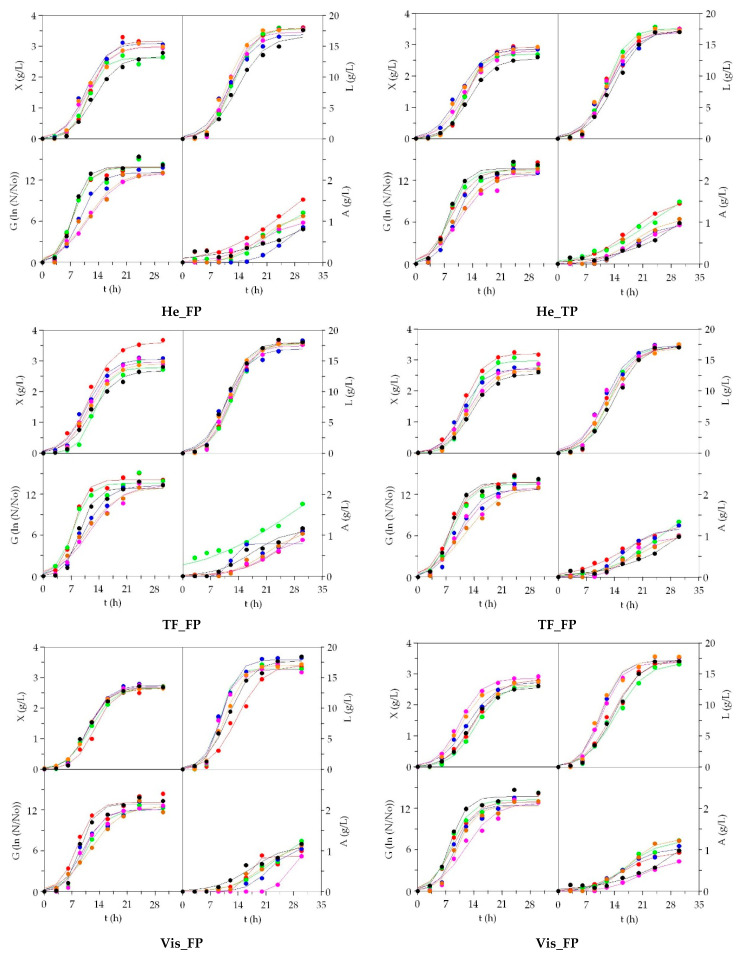
Fermentations of *L. plantarum* in low-cost media based on peptones from aquaculture by-products. ●: salmon; ●: trout; ●: turbot; ●: seabream; ●: seabass; ●: Man, Rogosa and Sharpe medium (MRS). Experimental data of biomass (X), viable cells (G), lactic acid (L) and acetic acid (A) were fitted to the logistic equation. The confidence intervals of experimental data (for two replicates) were in all cases less than 20% of the experimental mean values and omitted for clarity.

The time-course of growth and metabolic productions of *L. plantarum* in all media tested, including commercial MRS are displayed in Figure 2. Moreover, pH kinetics and nutrients uptakes (soluble protein and reducing sugars) were also recorded but not shown in the manuscript. The pH-tendencies for both LAB were completely similar in all cultivations showing conventional decreasing logistic patterns with non-null asymptote [48]. The uptakes of reducing sugars were almost exhaustive at the end of the cultures; however, Pr total consumptions were always lower than 2.5 g/L.

Experimental data of *L. plantarum* bioproductions were accurately predicted by Equation (1) including the complete description of kinetic phases derived from Equations (2)–(4). Tables S2 and S3 compile the numerical values of the mentioned parameters. The correlations between experimental and theoretical data were excellent: R^2^ ranged 0.989–0.999 for *X*, 0.967–0.991 for *G*, 0.974–0.998 for *L* and 0.939–1.000 for *A*. All parameters obtained from biomass, cells and lactic acid kinetics were statistically significant (for α = 0.05, t-Student test), but some of them from acetic acid were not. The consistency of fittings was, for all cultures, confirmed by the F-Fisher test (*p* < 0.005).

In aquaculture media the bioproductions were always similar or higher than those generated by MRS; the simple view of the kinetics indicate these trends (Figure 2). There were no notable differences between the type of processing performed to obtain the peptones. The higher values of maximum biomass (*X_m_*) was found in Sa_TF_FP (3.61 g/L) followed by Sa_TF_TP, Sa_He_FP, Tu_He and Tu_TF, all of them statistically superior to control medium (2.68 g/L). The rate parameters for *L. plantarum* growth (*v_x_, μ_x_*) and the time-dependent coefficients (*λ_x_*, *τ_x_*, *t_mx_*) were almost always statistically similar in the different nutritive broths assessed. The growth, in terms of cell parameters, showed identical behaviors (largest in salmon derived peptones), but without significant numerical differences comparing media to MRS (*p* > 0.05). Lactic acid maximum (*L_m_*) productions were also statistically equal in all cultures, although differences of 1.7 g/L were reached among Sa_He_FP and Sb_Vis_FP. The values of production rates and the time-dependent coefficients (*λ_L_*, *τ_L_*, *t_mL_*) followed similar patterns than exposed by growth. In many fermentations, the parameters for acetic acid (*A_m_*, *v_A_*, *λ_A_*, etc.) could not be determined because no clear asymptotic phase and logistical shapes were obtained. The real experimental data of acetic acid were produced in the interval of 0.75–1.50 g/L (Figure 2).

Regarding *L. brevis*, Figure S1 shows its corresponding experimental results and fitting profiles of *X, G, L* and *A* dependent-variables to the logistic model (1). This equation was an adequate tool to simulate the *L. brevis* time-course bioproductions: the determination coefficients ranging 0.952–1.000, and consistency and robustness of equations were also confirmed (*p*-values < 0.005). The values of *X_m_* were numerically larger in media including hydrolyzed peptones from He and TF of salmon than that reported by MRS. Nevertheless, the differences between alternative and control media were practically non-existent (*p* > 0.05), including some case with maximum biomass below 4 g/L. In general, TP led to slightly higher biomass production than FP (Tables S4 and S5). There were also no differences among the media for the biomass rates and time-depending parameters, except in Sb_Vis_FP, RT_Vis_FP and Sbass_TP where the rates were lower and the times were longer.

Lack of statistical significance, between aquaculture peptone media and MRS, was found for the production of viable cells (*G_m_*) and their associated parameters (*p* > 0.05). The lactic acid concentrations (values of *L_m_*) were higher in TP than FP, achieving 18.7 g/L in Sa_TF_TP and only 15.4 g/L in RT_He_FP, but the production rates showed contrary results. Nevertheless, in most of fermentations significant differences were almost not observed among broths and MRS (*p* > 0.05). However, the data of acetic acid were slightly higher in FP media with low differences in the two fermentations run as controls in MRS. Again, rate coefficients were identical, in statistical terms, in all media in relation to the commercial one.

The minima and maxima values of the productive yields for LAB, together with the media in where they were obtained, are summarized in Table S6. According these outcomes, *L. brevis* was the most efficient lactobacilli in the production of biomass as dry weight and acetic acid per unit of sugar and soluble protein. In return, *L. plantarum* yielded higher productivities for lactic acid and viable cells. The alternative media formulated with Sa_TF_FP showed the highest yields in many fermentations (*Y_X/RS_*_,_ *Y_X/Pr_*_,_ *Y_G/RS_* and *Y_G/Pr_* in *L. plantarum* and *Y_X/RS_* and *Y_G/Pr_* in *L. brevis*), MRS was the best option for the production of lactic acid in both strains, and RT_TF (from TP and FP) was better in the formation of acetic acid.

Summarizing, it can indicate that aquaculture peptones recovered by thermal or enzymatic procedure are excellent substitutes of meat extract and bactopeptone to ferment LAB. Although, FP and TP have differences in the composition of amino acids (Table 2 and Table 3), they are adequately balanced, including essential ones, for the growth and metabolite production from LAB [40,41]. The present results are in agreement with the capacity of other marine protein sources to cultivate *Lactobacilli* [37,48,49]. On the other hand, the use of peptones from marine origin have revealed the importance of the type of peptones present in the culture media for promoting the production of bacteriocins by LAB, since peptones from snow crab [23], tilapia viscera [20], squid and trout viscera [25] and tuna viscera [33] have conducted to important increasing of divergicin, nisin and pediocin formation. Further studies should be carried out to demonstrate if this tendency is also observed with aquaculture peptones.

### 3.3. Growth of Marine Probiotic Bacteria on Media Formulated with Peptones from Aquaculture Wastes

The medium commonly used for the growth of marine bacteria is a specific broth called Marine medium, which is a low nutritive formulation based on various minerals salts (in order to simulate seawater) and two protein ingredients (yeast extract and a commercial peptone). In this particular case, a simple alternative formulation composed by filtrate seawater, yeast extract and the aquaculture peptones was tested. Regarding microorganisms, *Phaeobacter* sp. and *P. fluorescens* are two marine bacteria that were shown to have probiotic properties in aquaculture diseases [50,51,52]. They must be produced to large scale for its potential application in the control of fish mortalities and, therefore, cost-effective media are necessary to generate huge amount of biomass and viable cells of those probiotic bacteria.

**Figure 3:**
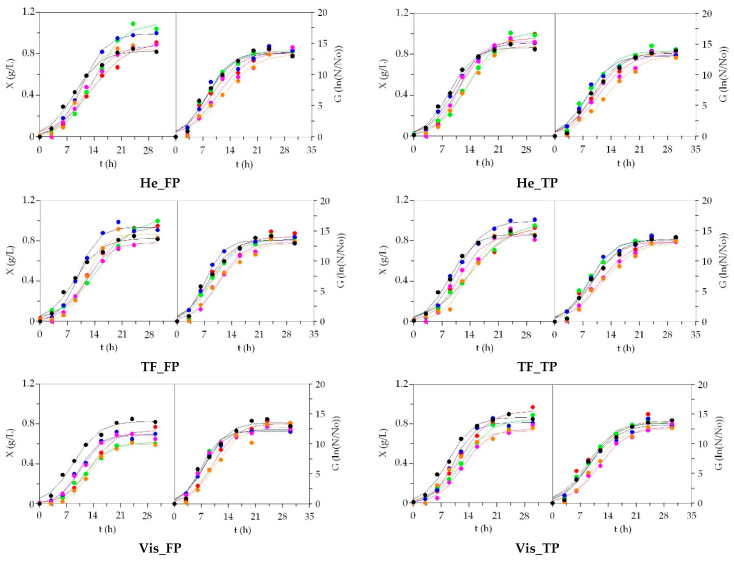
Growth of *Phaeobacter* sp. in low-cost media based on peptones from aquaculture by-products. ●: salmon; ●: trout; ●: turbot; ●: seabream; ●: seabass; ●: MRS. Experimental data of biomass (X) and viable cells (G) were fitted to the logistic equation. The confidence intervals of experimental data (for two replicates) were in all cases less than 20% of the experimental mean values and omitted for clarity.

Experimental data and predicted curves of *Phaeobacter* sp. growth in the alternative and control media are represented in Figure 3. The production profiles were very similar for all media, mainly for the case of cell formation. As in LAB, the correspondence between real data and logistical profiles was high (R^2^: 0.976–0.999 for *X* and 0.962–0.992 for *G*), the fittings consistencies were established but not all parametric estimates were significant (cell latencies were in many situations unclear). Alternative media including peptone of RT_He_FP led to the largest maximum dry weight meanwhile RT_Vis_FP, together with Sbass_Vis_FP, produced the lowest ones (Tables S7 and S8). In both cases, the differences in comparison with MM results were statistically significant (*p* < 0.05). Nevertheless, in global terms, no differences are observed among FP and TP peptones. No clear tendencies were found for biomass rate estimations. The formation of viable cells was statistically equal in all media and significant differences were not found (*p* > 0.05). Identical findings were defined for the rest of the numerical estimates of cell growth (rates and time-depending coefficients).

In productive terms, the yields of biomass (*Y_X/Pr_*) and cell generation (*Y_G/Pr_*) of *Phaeobacter* sp. per soluble protein consumption was always higher in the Marine commercial medium, being RT_He_FP and Tu_He_FP for *X* and Sbass_Vis and RT_Vis for *G*, the most effective aquaculture media.

As in the previous marine bacteria, the predictive ability of Equation (1) to describe the experimental data of *P. fluorescens* was remarkable (Figure S2) with determination coefficients always greater than 0.926 for biomass and 0.882 for cells (Tables S9 and S10). Because of the experimental trends were not perfectly sigmoidal (latency phases were non-existent and some outlier points were found at the end of the cultures), the confidence intervals of many *X_m_* and *G_m_* were very large and the estimates of *λ_X_* and *λ_G_* were not significant in almost all cultivations (t-Student test). Although numerically the highest maximum biomass and viable cells were achieved in MM, all media yielded similar production capacity. The growth rates, in both biomass and cells, were also statistically indistinguishable with those promoting by commercial medium.

Finally, the efficiency of MM in order to metabolize the soluble protein incorporated in culture medium (values of *Y_X/Pr_* and *Y_G/Pr_*) was superior to the alternative media. This efficiency was also higher for FP than TP. Aquaculture peptones have demonstrated to be an excellent protein ingredient for the growth of MPB in a similar way as was reported for peptones recovered from swordfish and tuna viscera and the production of marine chitin [48,53].

### 3.4. Growth of Aerobic Gram+ Bacteria Using Aquaculture Peptones

**Figure 4:**
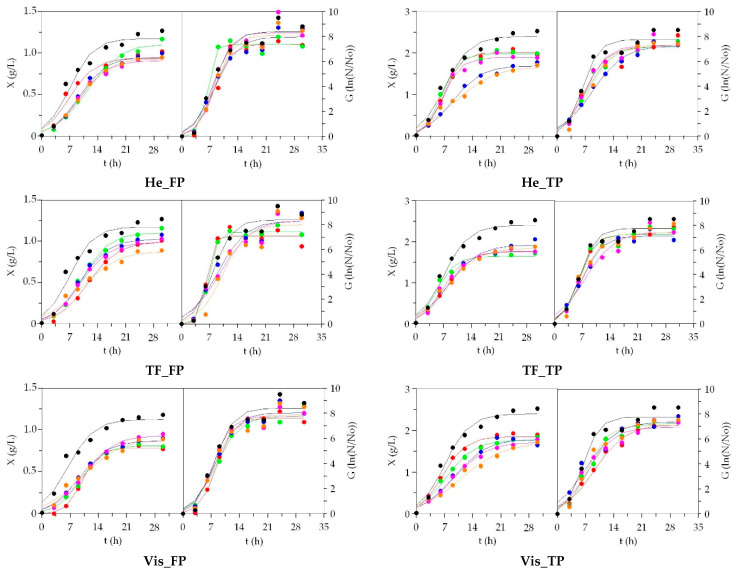
Growth of *S. epidermidis* in low-cost media based on peptones from aquaculture by-products. ●: salmon; ●: trout; ●: turbot; ●: seabream; ●: seabass; ●: MRS. Experimental data of biomass (X) and viable cells (G) were fitted to the logistic equation. The confidence intervals of experimental data (for two replicates) were in all cases less than 20% of the experimental mean values and omitted for clarity.

One of the most employed complex mediums for the culture of bacteria is tryptone soy broth (TSB). It is a nutritive preparation recommended for an ample number of aerobic strains including genus *Escherichia*, *Bacillus* and *Streptococcus*. It is composed of two mineral salts, two peptones (from soy and casein hydrolysis) and a low level of glucose. The high concentration of protein in relation to sugar makes TSB an ideal candidate to study the validity of aquaculture peptones as substitutes of the commercial ones. Moreover, *S. epidermidis* and *B. subtilis* are ubiquitous and very common bacteria in human skin and soil and are extensively quantified and manipulated in labs of microbiology throughout the world. The cultivation kinetics of both bacteria, in terms of biomass and cell production, are shown in Figure 4 and Figure S3, respectively. The time-course of pH, glucose and protein uptakes in all media was not displayed, but experimental shapes were rather similar in the different broths (the consumption of glucose was exhaustive and no more of 3 g/L was metabolized by *S. epidermidis* and *B. subtilis*).

From the point of view of mathematical fitting, Equation (1) remarkably simulated the experimental data of both bacterial biomass (R^2^ > 0.948) and slightly lowered the cell production (R^2^ > 0.930). The growth kinetic did not follow perfect sigmoidal forms since latencies phases were not observed on many occasions. Thus, the lag-phase values (*λ_X_* and *λ_G_*) were not significant in those situations. Some outlier data of cells were also found at asymptotic phase of cells (Figure 4 and Figure S3), but they were included in the procedure of modeling. Nevertheless, the robustness of the equation was always observed in all kinetics (*p* < 0.05 from F-Fisher test).

In *B. subtilis*, the greatest maxima biomass and cell productions were found in commercial TSB medium, although the differences with many alternative media were not statistically significant (Table S11). Comparing wastes, viscera TP peptones showed slightly lower bioproductions, but the process to generate peptones did not influence the competence of aquaculture peptones to grow *B. subtilis* (Tables S11 and S12). For the rest of parameters, no differences were observed between the tested media. Identical findings were achieved in *S. epidermidis*. TSB was the best option (in terms of *X_m_* values) with significant improving in relation to the alternative media (Tables S13 and S14). Sa_He was the finest option among low-cost media. Again, the rate and time-dependent parameters were statistically similar in all cases and both types of peptones did not show remarkable differences to support *S. epidermidis* fermentation. In general, the yields for biomass (as dry weight) and cell formation of both bacteria, in relation to sugars and protein consumptions, were higher in the cultures of TSB than in the alternative media. Sb_TF, Sbass_TF, Sb_He or RT_He were in many situations the most efficient peptones from fish farming residues.

These highlights in the use of aquaculture peptones are in correspondence with other food wastes used as source of protein for the production of *B. subtilis* and *S. epidermidis* strains. Hydrolysates of hake wastes from fillets preparation were successfully utilized for the growth of several bacteria including *B. subtilis* and *S. epidermidis* [54]. Production of lipase by a strain of *S. epidermidis* isolated from crustacean viscera was maintained by including tuna food processing in culture broth [55]. On the other hand, peptones from waste chicken feathers [56] and ray viscera [57] yielded excellent capacity for the growth of *B. subtilis*. The values of biomass achieved in those reports were lower than those obtained in the present work. The production of different enzymes, as protease and keratinase, by *B. subtilis* were supported in media formulated with fish substrates [58] and vegetal waste fractions [59,60].

### 3.5. Simple Economical Evaluation of Aquaculture Peptones for Bacterial Bioproductions

**Figure 5:**
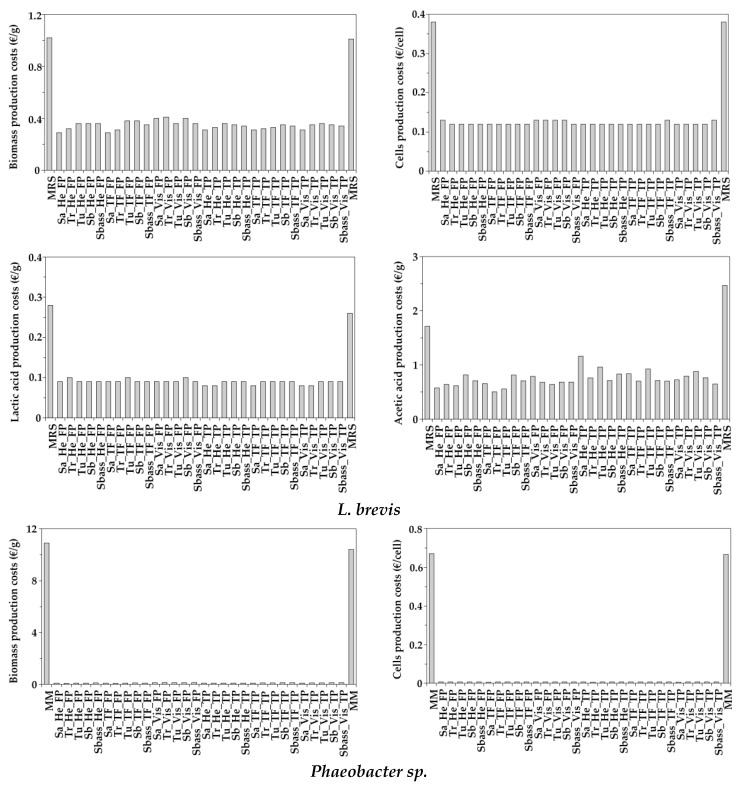
Economical evaluation of *L. brevis* and *Phaeobacter* sp. bioproduction costs in the nutritive media studied.

The cost of biomass, viable cells, lactic and acetic acids productions for *L. brevis* could be reduced at least three-four folds when aquaculture peptones were present in alternative MRS media in comparison to commercial MRS (Figure 5). Similar results were also observed for *L. plantarum* (Figure S4) with more than 200% of bioproduction depletion costs. Some histograms for acetic acid were not able to be displayed (Figure S4) because the corresponding *A_m_* parameters were not statistically significant (Tables S5 and S6). In the case of MPB, the range of reduction varied from 72 to 133 times (for *Phaeobacter* sp.) and from 74 to 122 (for *P. fluorescens*) in the alternative marine media that included seawater and peptones from aquaculture by-products. For *B. subtilis* (Figure S5), the savings observed ranging 16–22 and 22–26 folds in terms of biomass and cells, respectively. In a similar magnitude, Se reached reductions between 15–20 times (using *X_m_*) and 20–23 times (using *G_m_*).

These economical findings were in concordance with the results obtained when effluents of chitin production from endoskeletons of squid were utilized as a source of organic nitrogen in the culture of bacteria [48]. Furthermore, the high concentration of biomass and viable cells of probiotic bacteria generated together with the level of lactic acid produced in aquaculture peptones, along with the low cost of production, could help to improve cost benefits of the fish farming industry. The current proposal is also in agreement with postulates defined by the so-called circular economy [61,62,63].

## 4. Conclusions

In this study, the sustainable valorization of aquaculture wastes generated from food filleting by microbial bioconversion was first explored. Initially, two types of peptones were recovered from residues (head, viscera and trimmings + frames) of salmon, trout, turbot, seabream and seabass. Thus, 30 aquaculture peptones were incorporated in cost-effective media as protein substrates in the replacing of corresponding commercial peptones present in MRS, MM and TSB media. Several bacteria from different origins, genus and technological properties were cultivated in those media and the fermentation kinetics were adequately modeled by logistic equation. In all cases, aquaculture peptones supported the growth in similar or a higher extension than control media and, in economical terms, a remarkable reduction of bioproduction costs was also reported: specifically around 3–4, 70–130 and 16–26 folds in LAB, MPB and ubiquitous Gram (+) bacteria, respectively. Although this biotech strategy showed excellent results, further estimations of life cycle assessment (LCA) and CO_2_-footprint must be performed to confirm the complete validity of this study in comparison with other valorization protocols.

## Figures and Tables

**Table 1:** Basic biochemical composition of aquaculture peptones (mean values ± confidence intervals) obtained from enzyme (FP) and thermal processing (TP). Pr: Total soluble protein; N: Total nitrogen; RS: Reducing sugars; TS: Total sugars. He, TF, and Vis mean head, trimmings + frames and viscera, respectively. Sa: salmon, RT: rainbow trout, Tu: turbot; Sb: seabream; Sbass: seabass.

FP Peptones	Pr (g/L)	N (g/L)	RS (g/L)	TS (g/L)	TP Peptones	Pr (g/L)	N (g/L)	RS (g/L)	TS (g/L)
**Sa_He**	61.0 ± 1.3	14.6 ± 2.1	0.19 ± 0.04	1.29 ± 0.09	**Sa_He**	47.9 ± 1.2	11.3 ± 0.3	0.10 ± 0.01	0.60 ± 0.03
**Sa_TF**	69.7 ± 2.1	15.9 ± 1.4	0.27 ± 0.10	1.50 ± 0.10	**Sa_TF**	36.8 ± 0.7	8.6 ± 0.4	0.12 ± 0.04	0.80 ± 0.10
**Sa_Vis**	42.7 ± 2.2	10.0 ± 0.8	0.44 ± 0.18	1.37 ± 0.14	**Sa_Vis**	30.3 ± 1.4	7.2 ± 0.5	0.24 ± 0.07	1.17 ± 0.09
**RT_He**	47.8 ± 4.8	14.1 ± 1.1	0.21 ± 0.05	1.40 ± 0.10	**RT_He**	36.3 ± 1.4	10.4 ± 0.7	0.13 ± 0.01	0.97 ± 0.07
**RT_TF**	53.9 ± 5.1	14.7 ± 0.6	0.20 ± 0.02	1.22 ± 0.10	**RT_TF**	19.6 ± 0.3	5.4 ± 0.3	0.05 ± 0.02	0.52 ± 0.04
**RT_Vis**	50.6 ± 3.2	14.4 ± 0.9	0.16 ± 0.06	1.09 ± 0.15	**RT_Vis**	34.3 ± 1.6	9.5 ± 1.0	0.31 ± 0.02	1.40 ± 0.06
**Tu_He**	73.5 ± 4.9	18.4 ± 1.0	0.38 ± 0.06	1.26 ± 0.14	**Tu_He**	73.1 ± 1.5	18.6 ± 0.6	0.19 ± 0.04	1.29 ± 0.10
**Tu_TF**	73.9 ± 3.8	18.5 ± 0.7	0.36 ± 0.04	1.34 ± 0.17	**Tu_TF**	63.6 ± 3.7	15.9 ± 0.8	0.22 ± 0.05	1.36 ± 0.11
**Tu_Vis**	61.6 ± 2.8	15.4 ± 1.1	0.30 ± 0.09	1.39 ± 0.25	**Tu_Vis**	36.6 ± 2.3	9.2 ± 0.6	0.13 ± 0.06	1.52 ± 0.03
**Sb_He**	61.6 ± 1.6	17.6 ± 0.8	0.49 ± 0.06	1.78 ± 0.12	**Sb_He**	28.2 ± 2.1	9.0 ± 0.3	0.06 ± 0.00	0.72 ± 0.40
**Sb_TF**	81.2 ± 3.1	23.2 ± 1.3	0.32 ± 0.07	1.31 ± 0.07	**Sb_TF**	18.8 ± 0.2	6.3 ± 0.6	0.31 ± 0.09	1.24 ± 0.06
**Sb_Vis**	37.9 ± 1.7	12.1 ± 0.6	0.09 ± 0.01	0.84 ± 0.09	**Sb_Vis**	28.0 ± 1.4	8.9 ± 0.5	0.24 ± 0.07	1.67 ± 0.09
**Sbass_He**	63.3 ± 0.4	18.2 ± 0.8	0.35 ± 0.05	1.42 ± 0.12	**Sbass_He**	30.6 ± 0.2	8.5 ± 0.3	0.17 ± 0.05	0.88 ± 0.10
**Sbass_TF**	78.9 ± 5.1	20.8 ± 1.3	0.43 ± 0.08	1.67 ± 0.16	**Sbass_TF**	20.1 ± 2.3	5.8 ± 0.6	0.10 ± 0.03	0.80 ± 0.08
**Sbass_Vis**	33.0 ± 1.6	9.2 ± 0.5	0.08 ± 0.02	0.94 ± 0.07	**Sbass_Vis**	18.6 ± 0.6	5.6 ± 0.3	0.08 ± 0.01	0.56 ± 0.11

**Table 2:** Amino acids content (% or g/100 g total amino acids) of aquaculture FPH peptones (FP) expressed as mean value ± confidence interval.

Amino Acids	Sa_He	Sa_TF	Sa_Vis	RT_He	RT_TF	RT_Vis	Tu_He	Tu_TF	Tu_Vis	Sb_He	Sb_TF	Sb_Vis	Sbass_He	Sbass_TF	Sbass_Vis
**Asp**	9.61 ± 0.30	10.33 ± 0.06	9.89 ± 0.47	9.78 ± 0.19	10.32 ± 0.20	9.54 ± 0.07	8.81 ± 0.16	9.63 ± 0.10	9.26 ± 0.42	9.46 ± 0.03	10.80 ± 0.01	9.52 ± 0.00	9.25 ± 0.17	9.82 ± 0.03	9.69 ± 0.16
**Thr**	3.83 ± 0.39	2.95 ± 0.04	3.49 ± 1.15	4.38 ± 0.22	4.44 ± 0.15	5.30 ± 0.10	3.64 ± 0.16	3.90 ± 0.06	3.85 ± 0.18	4.52 ± 0.03	4.30 ± 0.07	4.63 ± 0.07	4.31 ± 0.05	4.48 ± 0.04	4.80 ± 0.03
**Ser**	4.98 ± 0.05	4.98 ± 0.11	5.02 ± 0.06	5.00 ± 0.20	4.83 ± 0.06	6.09 ± 0.03	5.67 ± 0.14	5.37 ± 0.18	5.39 ± 0.36	4.82 ± 0.01	4.68 ± 0.04	5.07 ± 0.07	4.89 ± 0.06	4.62 ± 0.02	5.60 ± 0.03
**Glu**	13.42 ± 0.45	13.23 ± 0.08	13.41 ± 0.65	13.89 ± 0.14	14.98 ± 0.34	13.05 ± 0.18	12.86 ± 0.18	13.55 ± 0.08	13.12 ± 0.32	13.83 ± 0.11	15.11 ± 0.11	12.91 ± 0.85	13.63 ± 0.07	14.21 ± 0.05	13.04 ± 0.21
**Gly**	12.49 ± 1.11	11.08 ± 0.27	11.66 ± 0.98	9.93 ± 1.10	8.94 ± 2.94	6.93 ± 0.06	14.50 ± 0.32	12.57 ± 0.15	13.00 ± 0.50	10.04 ± 0.08	8.63 ± 0.17	8.57 ± 0.01	10.84 ± 0.11	9.66 ± 0.01	7.75 ± 0.04
**Ala**	7.92 ± 0.45	8.45 ± 0.03	7.86 ± 1.01	7.19 ± 0.31	6.98 ± 0.22	7.17 ± 0.01	8.38 ± 0.24	8.06 ± 0.05	7.83 ± 0.22	7.39 ± 0.08	7.69 ± 0.15	7.23 ± 0.07	7.60 ± 0.01	7.56 ± 0.04	7.54 ± 0.01
**Cys**	0.75 ± 0.11	0.83 ± 0.03	0.99 ± 0.30	0.76 ± 0.09	0.74 ± 0.05	0.70 ± 0.02	0.62 ± 0.05	0.77 ± 0.10	0.71 ± 0.11	0.62 ± 0.01	0.89 ± 0.01	0.97 ± 0.09	0.69 ± 0.10	0.68 ± 0.05	0.96 ± 0.16
**Val**	3.39 ± 0.16	3.44 ± 0.30	3.37 ± 0.27	4.35 ± 0.33	4.24 ± 0.22	5.67 ± 0.14	2.96 ± 0.09	3.21 ± 0.11	3.23 ± 0.12	4.36 ± 0.01	3.70 ± 0.23	4.90 ± 0.02	4.23 ± 0.06	4.15 ± 0.03	5.54 ± 0.11
**Met**	3.13 ± 0.28	3.82 ± 0.33	3.45 ± 0.90	3.16 ± 0.10	3.33 ± 0.15	2.82 ± 0.14	2.76 ± 0.15	2.90 ± 0.04	2.84 ± 0.17	2.95 ± 0.14	3.33 ± 0.14	2.83 ± 0.05	2.76 ± 0.04	3.04 ± 0.01	2.62 ± 0.11
**Ile**	2.28 ± 0.22	2.02 ± 0.16	2.22 ± 0.28	3.22 ± 0.29	3.21 ± 0.23	4.48 ± 0.11	1.97 ± 0.11	2.19 ± 0.08	2.29 ± 0.12	3.38 ± 0.17	2.58 ± 0.04	3.65 ± 0.01	3.17 ± 0.04	3.40 ± 0.01	4.03 ± 0.22
**Leu**	6.17 ± 0.33	6.36 ± 0.22	6.27 ± 0.07	7.09 ± 0.33	7.19 ± 0.07	7.97 ± 0.15	5.37 ± 0.11	5.93 ± 0.11	5.86 ± 0.17	6.65 ± 0.14	7.07 ± 0.09	7.54 ± 0.01	6.31 ± 0.06	6.72 ± 0.03	7.75 ± 0.13
**Tyr**	3.37 ± 0.45	4.40 ± 0.17	3.88 ± 0.97	3.36 ± 0.18	3.39 ± 0.22	3.64 ± 0.26	2.90 ± 0.16	3.18 ± 0.08	3.11 ± 0.20	3.42 ± 0.02	3.59 ± 0.05	3.51 ± 0.08	3.34 ± 0.02	3.07 ± 0.02	3.99 ± 0.21
**Phe**	4.93 ± 0.89	7.15 ± 1.13	7.00 ± 3.41	4.38 ± 0.25	4.09 ± 0.15	4.55 ± 0.00	4.16 ± 0.18	4.51 ± 0.17	4.31 ± 0.37	4.03 ± 0.07	4.64 ± 0.12	4.93 ± 0.23	4.14 ± 0.09	3.98 ± 0.01	4.96 ± 0.15
**His**	2.00 ± 0.13	2.11 ± 0.17	2.10 ± 0.03	2.20 ± 0.49	2.18 ± 0.02	2.50 ± 0.02	1.61 ± 0.07	1.75 ± 0.04	1.85 ± 0.11	2.36 ± 0.05	2.67 ± 0.02	2.26 ± 0.01	2.07 ± 0.01	2.13 ± 0.01	2.08 ± 0.05
**Lys**	7.04 ± 0.46	7.96 ± 0.37	6.38 ± 0.33	7.78 ± 0.42	8.60 ± 0.13	6.97 ± 0.05	5.57 ± 0.14	6.15 ± 0.11	6.18 ± 0.23	7.13 ± 0.07	7.81 ± 0.12	7.62 ± 0.06	6.93 ± 0.11	8.05 ± 0.07	7.55 ± 0.01
**Arg**	5.69 ± 0.40	4.44 ± 0.03	5.18 ± 1.52	5.97 ± 0.10	5.96 ± 0.14	6.03 ± 0.01	6.53 ± 0.24	6.34 ± 0.08	6.59 ± 0.36	6.56 ± 0.15	5.73 ± 0.05	6.37 ± 0.06	6.39 ± 0.09	6.47 ± 0.02	5.28 ± 0.06
**OHPro**	2.85 ± 0.62	2.00 ± 0.14	2.36 ± 0.78	2.25 ± 0.37	1.86 ± 0.38	1.43 ± 0.22	4.00 ± 0.19	3.14 ± 0.18	3.42 ± 0.35	2.70 ± 0.05	1.92 ± 0.14	2.42 ± 0.69	3.18 ± 0.13	2.31 ± 0.13	1.85 ± 0.17
**Pro**	6.15 ± 0.83	4.45 ± 0.28	5.46 ± 1.67	5.30 ± 0.34	4.72 ± 0.16	5.18 ± 0.24	7.67 ± 0.25	6.86 ± 0.13	7.14 ± 0.37	5.79 ± 0.16	4.84 ± 0.02	5.06 ± 0.04	6.27 ± 0.10	5.66 ± 0.03	4.97 ± 0.40

**Table 3:** Amino acids content (% or g/100 g total amino acids) of aquaculture Thermal peptones (TP) expressed as mean value ± confidence interval.

Amino Acids	Sa_He	Sa_TF	Sa_Vis	RT_He	RT_TF	RT_Vis	Tu_He	Tu_TF	Tu_Vis	Sb_He	Sb_TF	Sb_Vis	Sbass_He	Sbass_TF	Sbass_Vis
**Asp**	6.68 ± 0.22	6.84 ± 0.30	6.69 ± 0.24	7.68 ± 0.12	7.17 ± 0.02	9.31 ± 0.17	9.51 ± 0.29	7.66 ± 0.08	8.56 ± 0.11	7.06 ± 0.03	6.52 ± 0.16	9.81 ± 0.24	7.67 ± 0.14	6.54 ± 0.20	8.95 ± 0.03
**Thr**	2.68 ± 0.06	2.80 ± 0.06	2.74 ± 0.16	3.45 ± 0.09	3.04 ± 0.13	5.14 ± 0.14	2.94 ± 0.11	3.09 ± 0.09	3.65 ± 0.07	3.20 ± 0.01	2.93 ± 0.01	5.14 ± 0.09	3.31 ± 0.01	2.81 ± 0.10	4.59 ± 0.20
**Ser**	4.21 ± 0.06	4.64 ± 0.01	4.45 ± 0.54	4.97 ± 0.12	5.17 ± 0.46	6.47 ± 0.09	6.16 ± 0.06	6.10 ± 0.06	6.24 ± 0.14	4.33 ± 0.25	3.82 ± 0.28	5.09 ± 0.25	4.59 ± 0.03	4.20 ± 0.06	5.25 ± 0.19
**Glu**	10.74 ± 0.43	11.52 ± 0.39	11.24 ± 0.57	12.35 ± 0.07	11.88 ± 1.05	13.11 ± 0.09	12.11 ± 0.01	12.16 ± 0.13	13.38 ± 0.44	12.28 ± 0.78	11.33 ± 0.15	13.30 ± 0.59	12.67 ± 0.18	11.99 ± 0.25	13.95 ± 0.04
**Gly**	21.42 ± 0.19	20.04 ± 0.18	20.21 ± 2.57	16.87 ± 0.22	19.07 ± 0.30	9.48 ± 0.05	17.59 ± 0.46	17.07 ± 0.27	14.92 ± 0.13	17.18 ± 0.06	19.61 ± 0.13	7.49 ± 0.20	16.25 ± 0.25	19.59 ± 0.06	9.40 ± 0.27
**Ala**	9.03 ± 0.13	9.14 ± 0.42	9.09 ± 0.01	7.99 ± 0.22	8.25 ± 0.63	6.73 ± 0.06	10.05 ± 0.40	9.86 ± 0.27	9.59 ± 0.09	9.07 ± 0.02	9.44 ± 0.12	6.91 ± 0.01	9.35 ± 0.18	10.25 ± 0.10	7.76 ± 0.02
**Cys**	0.85 ± 0.07	0.41 ± 0.05	0.71 ± 0.35	0.52 ± 0.06	0.61 ± 0.24	0.57 ± 0.04	0.39 ± 0.07	0.47 ± 0.04	0.65 ± 0.04	0.86 ± 0.16	1.17 ± 0.06	1.35 ± 0.30	0.85 ± 0.12	0.91 ± 0.13	1.45 ± 0.02
**Val**	2.39 ± 0.06	2.53 ± 0.09	2.48 ± 0.11	3.19 ± 0.05	2.96 ± 0.19	4.96 ± 0.04	2.21 ± 0.05	2.33 ± 0.04	3.05 ± 0.14	2.88 ± 0.28	3.24 ± 0.38	5.63 ± 0.16	2.93 ± 0.15	2.25 ± 0.12	4.95 ± 0.02
**Met**	2.36 ± 0.03	2.66 ± 0.19	2.56 ± 0.37	2.54 ± 0.10	2.55 ± 0.17	2.79 ± 0.09	2.62 ± 0.12	2.71 ± 0.11	2.26 ± 0.07	2.51 ± 0.26	2.23 ± 0.18	2.58 ± 0.08	2.40 ± 0.10	2.13 ± 0.01	2.53 ± 0.16
**Ile**	1.47 ± 0.07	1.50 ± 0.15	1.52 ± 0.04	2.25 ± 0.15	1.74 ± 0.11	3.89 ± 0.11	1.37 ± 0.18	1.40 ± 0.08	1.66 ± 0.08	1.97 ± 0.14	1.30 ± 0.01	3.85 ± 0.25	1.80 ± 0.04	1.47 ± 0.20	3.03 ± 0.11
**Leu**	3.18 ± 0.06	3.08 ± 0.02	3.17 ± 0.09	4.51 ± 0.18	3.50 ± 0.28	6.61 ± 0.12	3.91 ± 0.15	3.98 ± 0.03	4.80 ± 0.05	4.63 ± 0.07	3.95 ± 0.14	7.32 ± 0.04	4.54 ± 0.01	3.61 ± 0.13	6.45 ± 0.17
**Tyr**	1.07 ± 0.06	0.97 ± 0.09	0.99 ± 0.09	1.56 ± 0.10	1.35 ± 0.26	2.92 ± 0.08	1.87 ± 0.12	1.92 ± 0.14	2.34 ± 0.06	1.89 ± 0.33	1.46 ± 0.16	3.91 ± 0.14	2.00 ± 0.13	1.24 ± 0.10	3.61 ± 0.11
**Phe**	3.19 ± 0.13	3.23 ± 0.02	3.33 ± 0.15	3.56 ± 0.05	3.47 ± 0.13	3.43 ± 0.06	3.27 ± 0.20	3.44 ± 0.06	3.38 ± 0.08	3.10 ± 0.47	2.73 ± 0.01	4.34 ± 0.09	3.49 ± 0.36	3.08 ± 0.16	4.29 ± 0.44
**His**	1.49 ± 0.01	1.58 ± 0.09	1.56 ± 0.16	1.89 ± 0.08	1.52 ± 0.21	2.47 ± 0.13	1.25 ± 0.06	1.33 ± 0.14	1.53 ± 0.06	1.69 ± 0.02	2.25 ± 0.05	2.62 ± 0.04	1.57 ± 0.03	1.25 ± 0.02	2.14 ± 0.04
**Lys**	6.39 ± 0.14	5.21 ± 2.39	6.35 ± 0.23	6.09 ± 0.21	6.77 ± 0.08	7.14 ± 0.08	4.47 ± 0.18	4.58 ± 0.12	5.13 ± 0.10	5.17 ± 0.12	6.12 ± 0.18	8.02 ± 0.09	5.68 ± 0.09	5.44 ± 0.08	7.88 ± 0.07
**Arg**	7.09 ± 0.11	7.25 ± 0.14	7.23 ± 0.16	7.00 ± 0.09	6.73 ± 0.25	6.93 ± 0.38	6.64 ± 0.10	6.72 ± 0.11	6.28 ± 0.07	6.79 ± 0.13	7.05 ± 0.05	6.13 ± 0.01	6.60 ± 0.13	6.52 ± 0.02	5.41 ± 0.04
**OHPro**	6.78 ± 0.56	7.06 ± 0.11	6.68 ± 0.37	5.76 ± 0.17	6.54 ± 0.06	2.31 ± 0.11	6.29 ± 0.18	6.00 ± 0.11	4.69 ± 0.18	6.29 ± 0.17	6.42 ± 0.43	1.58 ± 0.21	5.76 ± 0.71	7.26 ± 0.83	2.32 ± 0.34
**Pro**	8.97 ± 0.43	9.55 ± 0.37	8.99 ± 0.48	7.81 ± 0.21	7.67 ± 0.21	5.72 ± 0.05	9.35 ± 0.25	9.17 ± 0.10	7.89 ± 0.00	9.10 ± 0.09	8.42 ± 0.50	4.92 ± 0.26	8.55 ± 0.29	9.46 ± 0.41	6.03 ± 0.14

## Supplementary Materials

The following are available online at https://www.mdpi.com/2218-273X/10/8/1184/s1, Figure S1: Fermentations of L. brevis, Figure S2: Kinetic growth of P. fluorescens, Figure S3: Kinetic growth of B. subtilis, Figure S4: Economical evaluation of L. plantarum bioproduction costs, Figure S5: Economical evaluation of P. fluorescens, B. subtilis and S. epidermidis growth costs, Table S1: Composition of culture media, Table S2: Kinetic Parameters of L. plantarum cultures on FP media, Table S3: Kinetic Parameters of L. plantarum cultures on TP media, Table S4: Kinetic parameters of L. brevis cultures on FP media, Table S5: Kinetic parameters of L. brevis cultures on TP media, Table S6: Productive yields of LAB bioproductions, Table S7: Kinetic parameters of P. fluorescens cultures on FP media, Table S8: Kinetic parameters of P. fluorescens cultures on TP media, Table S9: Kinetic parameters of Phaeobacter sp. cultures on FP media, Table S10: Kinetic parameters of Phaeobacter sp. cultures on TP media, Table S11: Kinetic parameters of B. subtilis cultures on FP media, Table S12: Kinetic parameters of B. subtilis cultures on TP media, Table S13: Kinetic parameters of S. epidermidis cultures on FP media, Table S14: Kinetic parameters of S. epidermidis cultures on TP media.
