# Supplementary material for: Biotechnological Valorization of Food Marine Wastes: Microbial Productions on Peptones Obtained from Aquaculture By-Products
Source: Biomolecules. 2020 Aug 14;10(8):1184. doi: 10.3390/biom10081184 (PMC7465676; doi:10.3390/biom10081184)
Supplement: Supplementary file 1 [file biomolecules-10-01184-s001.pdf]

SUPPLEMENTARY MATERIAL

Biotechnological valorization of food marine wastes: Microbial productions on peptones obtained from aquaculture by-products

José Antonio Vázquez*, Ana I. Durán, Araceli Menduíña and Margarita Nogueira

Grupo de Reciclado y Valorización de Materiales Residuales (REVAL), Instituto de Investigaciones Marinas (IIM-CSIC), C/Eduardo Cabello, 6, CP 36208 Vigo, Galicia, Spain; jvazquez@iim.csic.es (J.A.V.); anais@iim.csic.es (A.I.D.); araceli@iim.csic.es (A.M.); marga@iim.csic.es (M.N.)

* Correspondence: jvazquez@iim.csic.es; Tel.: +34-986-231930 (J.A.V.)

Table S1. Composition of culture media, alternative ones based on aquaculture peptones and commercial ones, for bacterial productions (g/L).

INGREDIENTS	AM_MRS	MRS	AM_MM	MM	AM_TSB	TSB
**Glucose**	25.0	25.0	-	-	-	-
**Yeast extract**	4.0	4.0	-	-	-	-
**Sodium acetate**	5.0	5.0	-	-	-	-
**Ammonium citrate**	2.0	2.0	-	-	-	-
**K_2HPO_4**	2.0	2.0	-	-	-	-
**$MgSO_4$**	0.2	0.2	-	-	-	-
**$MnSO_4$**	0.05	0.05	-	-	-	-
**Tween 80**	1.0	1.0	-	-	-	-
**Meat extract**	-	8.0	-	-	-	-
**Bactopeptone**	-	10.0	-	-	-	-
**Aquaculture peptone as protein-Lowry**	10.0	-	-	-	-	-
**Ferric citrate**	-	-	-	0.10	-	-
**Sodium chloride**	-	-	-	19.45	-	-
**Magnesium chloride**	-	-	-	5.90	-	-
**Sodium sulphate**	-	-	-	3.24	-	-
**Calcium chloride**	-	-	-	1.80	-	-
**Potassium chloride**	-	-	-	0.55	-	-
**Sodium bicarbonate**	-	-	-	0.16	-	-
**Potassium bromide**	-	-	-	0.08	-	-
**Strontium chloride**	-	-	-	34.0 mg	-	-
**Boric acid**	-	-	-	22.0 mg	-	-
**Sodium silicate**	-	-	-	4.0 mg	-	-
**Sodium fluoride**	-	-	-	2.4 mg	-	-
**Ammonium nitrate**	-	-	-	1.6 mg	-	-
**Disodium phosphate**	-	-	-	8.0 mg	-	-
**Yeast extract**	-	-	1.0	1.0	-	-
**Peptone**	-	-	-	5.0	-	-
**Aquaculture peptone as protein-Lowry**	-	-	2.6	-	-	-
**Sea water (L)***	-	-	1.0	-	-	-
**Distilled water (L)****	-	-	-	1.0	-	-
**Glucose**	-	-	-	-	2.5	2.5
**K_2HPO_4**	-	-	-	-	2.5	2.5
**NaCl**	-	-	-	-	5.0	5.0
**Casitone**	-	-	-	-	-	17.0
**Soy peptone**	-	-	-	-	-	3.0
**Aquaculture peptone as protein-Lowry**	-	-	-	-	11.0	-

Alternative media (AM): formulated with the marine peptones obtained from aquaculture wastes. AM_MRS, AM_MM and AM_TSB to substitute MRS, MM and TSB, respectively.

*Volume of filtrated and sterilized sea water needed for residual media preparation.

**Volume of distilled water needed for commercial medium preparation.

Table S2. Numerical values and confidence intervals for parameters obtained from experimental data of *L. plantarum* predicted by equations (1-4) grown on alternative media formulated with FHP peptones (FP). R^2 is the determination coefficient among experimental and predicted data. NS: not significant. MRS was used as control commercial medium.

	**Sa_He**	**Sa_TF**	**Sa_Vis**	**RT_He**	**RT_TF**	**RT_Vis**	**Tu_He**	**Tu_TF**	**Tu_Vis**	**Sb_He**	**Sb_TF**	**Sb_Vis**	**Sbass_He**	**Sbass_TF**	**Sbass_Vis**	**MRS**
**Biomass (X)**																
X_m	3.16±0.20	3.61±0.33	2.65±0.28	2.62±0.16	2.78±0.19	2.68±0.23	3.10±0.18	3.07±0.17	2.75±0.22	2.99±0.19	2.97±0.24	2.71±0.17	3.00±0.22	2.91±0.30	2.65±0.11	2.68±0.19
v_m	0.35±0.10	0.29±0.09	0.27±0.19	0.31±0.10	0.34±0.10	0.23±0.07	0.21±0.08	0.27±0.09	0.27±0.11	0.26±0.09	0.27±0.10	0.26±0.10	0.26±0.08	0.25±0.07	0.26±0.11	0.26±0.08
λ_X	7.63±1.28	5.44±1.97	7.64±1.54	6.98±1.35	8.68±1.35	5.87±1.42	5.46±1.41	5.26±1.27	6.02±1.33	5.53±1.39	5.92±1.40	6.00±1.39	5.18±1.27	6.00±1.34	5.88±1.51	5.99±1.49
μ_X	0.44±0.13	0.32±0.11	0.40±0.13	0.48±0.15	0.48±0.16	0.35±0.16	0.38±0.14	0.35±0.15	0.39±0.14	0.35±0.12	0.36±0.14	0.39±0.15	0.35±0.12	0.38±0.14	0.39±0.13	0.39±0.10
τ_X	12.2±0.8	11.7±1.3	12.6±2.5	11.2±0.8	12.8±0.9	11.6±1.1	10.8±0.6	11.0±0.9	11.2±1.3	11.2±0.9	11.4±1.0	11.2±0.8	11.8±1.1	10.8±0.6	11.1±1.2	11.2±1.6
t_{mX}	16.7±1.8	17.9±3.0	17.6±2.4	15.4±1.8	17.0±1.9	17.4±1.9	16.1±2.4	16.8±2.2	16.3±1.9	16.9±1.8	17.0±1.8	16.3±1.8	17.6±2.2	16.1±2.4	16.3±2.5	16.3±2.0
R^2	0.994	0.989	0.993	0.993	0.993	0.997	0.989	0.991	0.994	0.991	0.993	0.994	0.993	0.989	0.993	0.993
**Cells (G)**																
G_m	13.9±0.9	14.1±0.8	13.1±2.0	13.8±1.3	13.6±1.2	12.1±1.0	13.1±1.4	13.3±1.5	12.1±1.2	13.0±1.1	13.3±1.0	12.0±1.4	13.2±1.4	13.0±1.2	12.6±1.2	12.9±1.3
v_G	1.67±0.58	2.04±0.68	1.52±0.60	1.62±0.76	1.64±0.78	1.10±0.51	1.35±0.81	1.13±0.77	1.16±0.63	0.86±0.50	0.96±0.43	1.32±0.62	0.88±0.36	0.84±0.39	0.86±0.31	1.73±0.80
λ_G	3.69±1.63	4.11±1.32	3.96±1.98	3.50±2.23	3.30±2.22	5.10±1.54	4.61±2.21	4.54±2.10	4.54±1.66	4.08±2.00	4.03±1.61	5.52±2.01	3.88±1.32	3.27±1.50	4.42±1.49	5.42±1.93
μ_G	0.48±0.18	0.58±0.20	0.46±0.30	0.47±0.23	0.48±0.24	0.37±0.13	0.41±0.20	0.34±0.18	0.38±0.15	0.26±0.19	0.26±0.17	0.44±0.19	0.27±0.15	0.26±0.18	0.27±0.19	0.54±0.21
τ_G	7.85±0.89	7.58±0.72	8.28±1.62	7.76±1.22	7.46±1.22	10.6±1.2	9.47±1.47	10.4±1.9	9.76±1.91	11.7±1.3	11.8±1.6	10.1±1.2	11.4±1.5	11.1±1.0	11.8±2.2	9.15±1.80
t_{mG}	12.0±1.9	11.0±1.5	12.6±3.0	12.0±2.6	11.6±2.6	16.1±3.3	14.3±3.0	16.3±3.6	15.0±2.7	19.3±4.0	19.6±3.7	14.7±2.2	18.9±3.4	18.8±2.8	19.1±3.9	12.9±2.8
R^2	0.989	0.991	0.971	0.979	0.978	0.983	0.986	0.983	0.967	0.990	0.978	0.982	0.975	0.979	0.985	0.985
**Lactic acid (L)**																
L_m	18.0±0.9	17.3±0.9	17.1±1.5	17.9±0.9	17.6±0.7	16.5±1.4	17.0±1.3	17.0±0.9	18.0±1.4	17.3±1.1	17.5±0.9	16.3±1.5	17.8±0.8	17.7±1.3	17.0±0.9	18.0±1.0
v_L	1.66±0.30	1.60±0.29	1.31±0.21	1.65±0.31	1.65±0.24	2.36±0.37	1.35±0.40	1.44±0.29	2.04±0.31	1.74±0.29	1.70±0.41	2.22±0.41	1.78±0.34	1.70±0.45	1.84±0.33	1.67±0.44
λ_L	6.92±1.03	7.31±1.03	7.31±1.51	7.13±1.06	6.72±0.82	5.89±1.07	5.42±1.09	5.17±0.97	5.45±1.26	6.70±1.19	6.66±1.30	5.85±1.32	6.30±1.22	6.20±1.18	6.10±1.16	5.86±2.00
μ_L	0.37±0.07	0.37±0.07	0.31±0.11	0.37±0.08	0.38±0.06	0.57±0.12	0.32±0.09	0.34±0.08	0.45±0.12	0.40±0.10	0.39±0.07	0.54±0.10	0.41±0.08	0.38±0.12	0.43±0.09	0.37±0.12
τ_L	12.3±0.7	12.7±0.7	13.8±1.5	12.6±0.7	12.0±0.5	9.39±1.14	11.7±0.9	11.1±1.3	9.86±1.4	11.7±1.0	11.8±1.2	9.53±0.97	11.2±1.6	11.4±1.3	10.7±0.9	11.3±1.8
t_{mL}	17.8±1.5	18.2±1.5	20.4±3.0	18.0±1.6	17.3±1.2	12.9±2.2	18.0±1.9	17.0±2.3	14.3±1.7	16.6±2.0	17.0±2.3	13.2±1.9	16.1±2.4	16.6±2.1	15.3±1.7	16.7±1.5
R^2	0.997	0.997	0.990	0.997	0.998	0.990	0.984	0.984	0.991	0.991	0.995	0.991	0.995	0.996	0.997	0.994
**Acetic acid (A)**																
A_m	2.42±2.00	2.22±2.03	0.87(NS)	1.33±0.47	3.23(NS)	1.49(NS)	1.03(NS)	0.7(NS)	1.12±0.74	1.07±1.01	1.01(NS)	0.87(NS)	1.17±0.21	1.15±0.25	1.24±1.10	1.17±0.91
v_A	0.08±0.03	0.09±0.05	0.18±0.16	0.08±0.03	0.07(NS)	0.08±0.06	0.09±0.05	0.21(NS)	0.10±0.04	0.05±0.03	0.06±0.04	0.30(NS)	0.08±0.03	0.05±0.02	0.07±0.06	0.06±0.05
λ_A	9.46±6.84	17.2±6.3	13.9±7.0	13.5±3.2	4.33(NS)	13.4±3.9	19.1±4.5	10.2(NS)	16.2±3.9	9.15±7.73	12.6±8.1	23.6(NS)	12.1±4.9	12.4±6.5	12.2±6.2	8.24±3.31
μ_A	0.12±0.07	0.16±0.07	0.81±0.74	0.25±0.15	0.08±0.08	0.21±0.17	0.33±0.21	1.07(NS)	0.36±0.24	0.20±0.19	0.23±0.15	1.17(NS)	0.27±0.09	0.23±0.20	0.24±0.19	0.22±0.10
τ_A	25.6±14.2	29.5±11.1	16.4±15.0	21.6±4.1	28.3(NS)	22.9±16.5	25.2(NS)	12.1(NS)	21.9±6.9	19.1±9.9	21.4(NS)	25.2(NS)	19.4±3.5	21.0±13.2	20.5±17.6	17.4±4.6
t_{mA}	41.7±22.5	41.8±16.2	18.9(NS)	29.6±8.5	52.3(NS)	32.4(NS)	31.2(NS)	14.0(NS)	27.5±5.8	29.1±7.3	30.2(NS)	26.8(NS)	26.8±5.1	29.6±12.4	28.9±16.0	26.6±3.9
R^2	0.975	0.991	0.949	0.971	0.939	0.982	0.998	0.886	0.992	0.971	0.988	1.000	0.996	0.978	0.981	0.963

Table S3. Numerical values and confidence intervals for parameters obtained from experimental data of *L. plantarum* predicted by equations (1-4) grown on alternative media formulated with thermal peptones (TP). R^2 is the determination coefficient among experimental and predicted data. NS: not significant. MRS was used as control commercial medium.

	Sa_He	Sa_TF	Sa_Vis	RT_He	RT_TF	RT_Vis	Tu_He	Tu_TF	Tu_Vis	Sb_He	Sb_TF	Sb_Vis	Sbass_He	Sbass_TF	Sbass_Vis	MRS
**Biomass (X)**																
X_m	2.92±0.09	3.21±0.20	2.82±0.12	2.70±0.12	2.97±0.17	2.64±0.10	2.86±0.08	2.71±0.11	2.75±0.09	2.81±0.07	2.76±0.09	2.85±0.11	2.85±0.10	2.67±0.07	2.64±0.12	2.56±0.10
v_m	0.31±0.10	0.31±0.12	0.22±0.11	0.33±0.10	0.34±0.11	0.22±0.10	0.25±0.08	0.26±0.10	0.26±0.09	0.23±0.08	0.23±0.08	0.26±0.09	0.26±0.08	0.22±0.09	0.25±0.11	0.22±0.10
λ_X	7.95±1.52	6.42±1.49	7.70±1.68	7.88±1.31	8.13±1.39	8.40±1.63	4.88±1.35	6.00±1.41	5.60±1.43	6.04±1.29	6.53±1.09	5.07±1.46	5.07±1.77	6.89±1.43	5.35±1.40	7.22±1.42
μ_X	0.43±0.15	0.39±0.16	0.32±0.14	0.49±0.14	0.46±0.15	0.33±0.13	0.34±0.11	0.39±0.12	0.30±0.12	0.33±0.12	0.34±0.11	0.37±0.12	0.37±0.11	0.34±0.12	0.38±0.13	0.34±0.10
τ_X	12.7±0.9	11.5±0.9	14.0±1.3	12.0±0.8	12.5±0.9	14.4±1.1	10.7±1.3	11.2±1.4	12.4±0.8	12.1±1.0	12.5±1.2	10.5±1.5	10.5±1.6	12.9±1.3	10.7±0.8	13.1±1.2
t_{mX}	17.4±1.8	16.6±2.1	20.3±2.5	16.1±1.7	16.8±1.9	20.5±1.7	16.6±1.8	16.3±1.8	19.2±2.1	18.1±1.7	18.5±2.0	16.0±1.9	16.0±1.4	18.8±1.5	16.0±1.6	19.0±2.2
R^2	0.999	0.997	0.998	0.999	0.997	0.998	0.992	0.994	0.995	0.992	0.992	0.997	0.997	0.999	0.992	0.998
**Cells (G)**																
G_m	13.6±0.9	13.8±0.9	12.9±1.3	13.4±1.3	13.5±1.2	13.2±1.2	12.9±1.3	12.7±1.4	12.4±2.1	12.8±1.7	13.0±1.1	12.8±1.6	13.3±1.2	13.0±1.1	12.3±1.6	13.7±1.8
v_G	1.22±0.59	1.37±0.63	1.19±0.59	1.56±0.72	1.41±0.66	1.37±0.66	1.38±0.60	1.13±0.54	1.44±0.57	0.89±0.58	0.91±0.60	0.90±0.51	0.96±0.54	0.77±0.63	1.41±0.69	1.70±0.69
λ_G	3.14±2.01	3.08±1.83	3.29±1.76	3.97±2.10	3.69±2.24	3.60±1.98	5.15±1.56	4.49±1.65	5.04±2.11	3.64±1.85	3.53±2.04	4.94±1.89	3.55±2.25	3.55±2.43	5.39±2.18	4.07±2.16
μ_G	0.36±0.21	0.40±0.19	0.37±0.24	0.47±0.19	0.42±0.18	0.41±0.20	0.43±0.22	0.36±0.24	0.46±0.19	0.28±0.21	0.28±0.15	0.28±0.23	0.29±0.16	0.24±0.22	0.46±0.16	0.50±0.24
τ_G	8.70±1.24	8.11±1.29	8.75±1.42	8.26±1.11	8.47±1.20	8.42±1.27	9.85±1.29	10.1±1.4	9.36±1.41	10.9±1.6	10.6±1.3	12.1±2.3	10.5±1.5	12.1±2.7	9.74±1.69	8.10±1.30
t_{mG}	14.3±3.0	13.1±3.1	14.2±2.5	12.6±2.3	13.2±2.0	13.2±2.2	14.6±3.0	15.7±2.9	13.7±2.6	18.1±3.3	17.8±2.9	19.2±3.5	17.4±3.3	20.6±3.9	14.1±2.6	12.1±3.6
R^2	0.982	0.980	0.975	0.988	0.981	0.983	0.987	0.977	0.979	0.982	0.975	0.974	0.988	0.980	0.985	0.990
**Lactic acid (L)**																
L_m	17.2±2.0	17.2±2.2	17.1±1.8	17.6±0.8	17.1±0.8	16.8±1.7	17.2±1.3	17.4±1.1	17.3±1.5	17.0±1.2	17.2±2.3	16.8±1.9	17.6±1.5	16.9±1.6	17.1±1.3	17.4±1.3
v_L	1.43±0.54	1.28±0.47	1.25±0.49	1.67±0.28	1.58±0.26	1.14±0.39	1.25±0.32	1.41±0.38	1.69±0.49	1.48±0.38	1.22±0.66	1.65±0.58	1.44±0.44	1.32±0.50	1.74±0.60	1.31±0.27
λ_L	6.57±2.23	6.17±2.57	6.62±2.32	6.84±0.91	7.22±0.94	6.96±1.77	5.91±1.43	5.57±1.59	5.58±1.87	6.60±1.69	5.02±2.45	5.78±1.90	6.10±2.01	6.29±1.97	5.51±1.99	7.28±1.43
μ_L	0.33±0.14	0.30±0.13	0.29±0.11	0.38±0.07	0.37±0.07	0.27±0.14	0.29±0.12	0.33±0.10	0.40±0.15	0.35±0.13	0.28±0.16	0.39±0.14	0.33±0.13	0.31±0.16	0.41±0.14	0.30±0.07
τ_L	12.6±1.6	12.9±1.9	13.5±2.0	12.1±0.6	12.6±0.9	14.3±2.9	12.8±2.0	11.7±2.4	10.6±2.8	12.3±3.0	12.1±2.9	10.9±2.5	12.2±1.8	12.7±2.6	10.4±2.2	14.0±1.1
t_{mL}	18.6±3.6	19.6±4.1	20.4±3.1	17.4±1.3	18.0±1.4	21.7±3.0	19.7±2.7	17.9±3.4	15.7±3.8	18.1±3.2	19.1±3.8	16.0±2.9	18.3±2.5	19.2±2.2	15.4±2.8	20.6±2.3
R^2	0.986	0.984	0.990	0.998	0.998	0.990	0.987	0.988	0.988	0.990	0.974	0.997	0.997	0.991	0.987	0.995
**Acetic acid (A)**																
A_m	1.57±0.49	1.33±0.40	0.96±0.42	1.95(NS)	1.89(NS)	1.22±0.51	0.96±0.13	1.18±0.19	1.04±0.31	1.04(NS)	0.96±0.41	0.77±0.71	1.11±0.71	11.4(NS)	1.27±0.31	3.54(NS)
v_A	0.08±0.05	0.06±0.03	0.06±0.02	0.07±0.05	0.08±0.06	0.08±0.03	0.07±0.02	0.09±0.03	0.07±0.03	0.06±0.05	0.08±0.03	0.05±0.02	0.07(NS)	0.06±0.03	0.09±0.03	0.10±0.08
λ_A	7.71(NS)	4.93(NS)	7.16±4.10	8.63(NS)	12.3±5.6	9.36±2.61	12.1±1.8	9.80±2.69	9.01±2.36	10.8±2.6	11.9±2.0	12.4±6.7	10.4±4.8	9.48±5.61	10.6±2.9	20.3(NS)
μ_A	0.20±0.08	0.17±0.09	0.23±0.06	0.15±0.11	0.16±0.10	0.26±0.06	0.29±0.08	0.30±0.11	0.27±0.09	0.21±0.13	0.35±0.09	0.25(NS)	0.24(NS)	0.19(NS)	0.29±0.15	0.11±0.06
τ_A	17.9±6.8	16.5±7.6	16.0±4.4	22.4(NS)	24.7(NS)	17.1±5.9	18.9±5.7	16.5±5.1	16.3±7.2	20.4±5.1	17.7±4.0	20.4±7.0	18.7±10.3	20.4(NS)	17.5±6.0	40.0(NS)
t_{mA}	28.0(NS)	28.2(NS)	24.8±7.0	36.3(NS)	37.1(NS)	24.9±8.5	25.7±7.2	23.1±7.9	23.6±8.8	30.0±24.8	23.5±10.1	28.4(NS)	27.0±23.1	30.9(NS)	24.3±8.8	60.2(NS)
R^2	0.993	0.986	0.996	0.984	0.994	0.989	0.993	0.983	0.980	0.985	0.983	0.994	0.948	0.987	0.993	0.974

Table S4. Numerical values and confidence intervals for parameters obtained from experimental data of *L. brevis* predicted by equations (1-4) grown on alternative media formulated with FHP peptones (FP). R^2 is the determination coefficient among experimental and predicted data. NS: not significant. MRS was used as control commercial medium.

	Sa_He	Sa_TF	Sa_Vis	RT_He	RT_TF	RT_Vis	Tu_He	Tu_TF	Tu_Vis	Sb_He	Sb_TF	Sb_Vis	Sbass_He	Sbass_TF	Sbass_Vis	MRS
**Biomass (X)**																
X_m	5.13±0.45	5.22±0.25	3.77±0.50	4.67±0.24	4.90±0.16	3.67±0.29	4.13±0.19	3.97±0.37	4.15±0.29	4.13±0.33	3.97±0.30	3.72±0.35	4.15±0.40	4.22±0.38	4.13±0.18	4.53±0.08
v_m	0.69±0.33	0.70±0.18	0.44±0.15	0.58±0.15	0.68±0.13	0.31±0.10	0.46±0.06	0.42±0.05	0.48±0.11	0.46±0.10	0.42±0.09	0.26±0.09	0.48±0.13	0.46±0.12	0.46±0.12	0.61±0.06
λ_X	5.25±2.02	7.22±1.05	6.19±0.95	5.55±1.16	6.03±0.74	7.41±1.74	5.27±0.83	4.95±1.31	5.54±1.60	5.27±1.52	4.95±1.76	7.18±1.63	5.54±1.59	5.30±1.30	5.27±0.98	6.65±0.40
μ_X	0.54±0.27	0.54±0.15	0.45±0.14	0.50±0.14	0.55±0.11	0.33±0.09	0.45±0.06	0.43±0.12	0.46±0.09	0.45±0.08	0.43±0.10	0.28±0.10	0.46±0.05	0.44±0.07	0.45±0.12	0.54±0.06
τ_X	8.98±1.08	11.0±0.6	10.5±1.4	9.56±0.63	9.65±0.40	13.4±1.1	9.72±0.79	9.65±0.99	9.86±1.13	9.72±1.10	9.65±1.03	14.3±1.2	9.58±0.96	9.92±1.25	9.72±0.59	10.4±0.2
t_{mX}	12.7±2.4	14.7±1.3	14.8±2.5	13.6±1.4	13.3±0.9	19.4±2.8	14.2±1.9	14.3±2.2	14.2±2.5	14.1±2.8	14.3±2.7	21.3±3.6	14.2±2.4	14.2±2.8	14.2±1.4	14.1±0.5
R^2	0.982	0.996	0.989	0.995	0.998	0.992	0.997	0.997	0.996	0.997	0.996	0.993	0.996	0.997	0.997	0.999
**Cells (G)**																
G_m	11.4±1.0	12.1±1.0	11.4±1.5	12.2±0.8	12.1±1.1	11.9±1.2	12.1±1.0	12.1±0.9	11.2±0.8	12.1±0.7	12.2±0.8	11.8±0.8	12.4±0.9	12.3±0.9	12.0±1.1	12.1±1.2
v_G	1.49±0.73	1.87±0.96	1.46±0.99	1.49±0.51	1.40±0.66	1.49±0.51	1.42±0.59	1.77±0.52	1.67±0.50	1.55±0.53	1.26±0.48	1.73±0.59	1.17±0.60	1.61±0.65	0.98±0.27	1.77±1.03
λ_G	4.30±2.11	5.06±1.88	5.65±1.59	4.95±1.57	4.09±2.24	4.47±2.21	4.51±1.41	4.89±1.69	4.35±1.35	4.65±1.16	3.62±0.97	4.65±1.52	3.64±1.32	3.18±0.99	4.27±1.90	4.93±2.24
μ_G	0.52±0.27	0.62±0.33	0.52±0.30	0.49±0.18	0.46±0.23	0.50±0.20	0.47±0.15	0.59±0.20	0.60±0.21	0.51±0.20	0.42±0.19	0.59±0.18	0.28±0.14	0.38±0.16	0.33±0.13	0.59±0.36
τ_G	8.14±1.14	8.28±0.99	9.54±1.32	9.06±0.85	8.41±1.23	8.45±1.10	8.76±0.91	8.29±0.78	7.71±0.63	8.54±1.06	8.43±1.16	8.04±0.96	8.47±0.97	8.44±1.24	10.4±0.9	8.35±1.19
t_{mG}	12.0±2.4	11.5±2.1	13.4±4.2	13.2±1.9	12.7±2.7	12.4±2.3	13.0±1.9	11.7±1.4	11.1±1.7	12.4±2.3	13.2±1.6	11.4±1.7	17.7±1.9	13.8±2.9	16.6±3.9	11.7±2.5
R^2	0.982	0.983	0.987	0.991	0.982	0.985	0.991	0.992	0.994	0.981	0.972	0.991	0.983	0.980	0.990	0.977
**Lactic acid (L)**																
L_m	16.3±0.5	16.3±0.6	16.0±0.8	15.4±0.4	15.8±0.6	16.3±0.4	16.2±1.1	15.4±1.2	16.1±0.5	17.2±0.3	17.3±0.4	14.6±0.9	17.1±0.5	16.9±0.5	16.6±0.5	16.3±0.9
v_L	2.00±0.31	1.78±0.32	1.82±0.28	1.90±0.24	1.90±0.33	2.23±0.18	2.11±0.50	1.86±0.49	2.29±0.29	1.97±0.19	1.89±0.21	2.24±0.13	2.04±0.18	1.89±0.17	1.83±0.72	1.81±0.16
λ_L	5.82±0.69	5.99±0.87	6.14±0.65	5.85±0.55	5.62±0.79	6.88±0.34	6.89±0.96	6.35±0.98	6.70±0.41	6.04±0.31	5.57±0.40	6.73±0.29	5.27±0.21	5.04±0.26	5.02±1.80	6.58±0.43
μ_L	0.49±0.08	0.44±0.08	0.46±0.12	0.50±0.07	0.48±0.09	0.65±0.14	0.52±0.13	0.49±0.17	0.57±0.06	0.46±0.07	0.44±0.08	0.61±0.07	0.48±0.09	0.45±0.06	0.44±0.15	0.44±0.04
τ_L	9.90±0.38	10.6±0.5	10.5±0.8	9.89±0.30	9.78±0.44	10.1±0.4	10.7±0.7	10.5±0.8	10.2±0.2	10.4±0.6	10.2±0.3	9.99±0.23	9.45±0.51	9.51±0.42	9.55±1.34	11.2±0.3
t_{mL}	14.0±0.9	15.1±1.1	14.9±1.8	13.9±0.7	13.9±1.0	13.2±0.5	14.5±1.6	14.6±2.0	13.8±0.4	14.8±0.5	14.7±0.5	13.3±0.6	13.6±0.6	14.0±0.4	14.1±3.0	15.8±0.6
R^2	0.998	0.998	0.999	0.999	0.998	0.999	0.992	0.991	0.999	0.994	0.993	1.000	0.996	0.995	0.989	0.999
**Acetic acid (A)**																
A_m	2.59±0.14	2.28±0.25	1.90±0.51	2.33±0.14	2.97±0.23	2.20±0.14	2.43±0.33	2.69±0.41	2.33±0.26	1.83±0.39	1.84±0.51	2.18±0.15	2.11±0.40	2.09±0.27	2.19±0.17	2.69±0.16
v_A	0.19±0.04	0.20±0.08	0.14±0.07	0.22±0.05	0.19±0.04	0.24±0.09	0.23±0.11	0.17±0.07	0.17±0.04	0.10±0.05	0.10±0.09	0.22±0.07	0.10±0.05	0.10±0.07	0.10±0.08	0.20±0.03
λ_A	4.22±1.24	6.25±2.34	8.02±1.58	7.16±1.24	3.97±1.63	6.66±1.71	7.31±2.51	6.31±2.61	6.25±2.16	6.41±1.59	10.1±2.9	6.23±1.50	8.54±2.60	9.63±1.93	6.13±1.80	5.73±1.18
μ_A	0.30±0.06	0.35±0.16	0.30±0.23	0.37±0.09	0.26±0.06	0.43±0.16	0.38±0.15	0.25±0.11	0.30±0.13	0.22±0.15	0.21±0.12	0.41±0.11	0.20±0.14	0.20±0.12	0.19±0.14	0.29±0.06
τ_A	10.9±0.8	11.9±1.5	14.8±9.9	12.6±0.8	11.8±1.2	11.3±0.9	12.6±1.8	14.2±1.9	13.0±1.7	15.6±1.8	19.6±2.7	11.1±0.8	18.7±3.0	15.1±1.6	17.0±2.6	12.6±0.9
t_{mA}	17.5±1.9	17.6±3.4	21.6±10.7	17.9±1.8	19.7±2.7	16.0±2.3	17.9±3.8	22.0±4.9	19.7±3.2	24.8±2.7	29.2±3.4	16.1±2.2	28.9±3.2	30.1±2.9	27.8±4.9	19.5±1.9
R^2	0.996	0.983	0.972	0.996	0.993	0.989	0.979	0.984	0.989	0.992	0.986	0.993	0.983	0.993	0.978	0.996

Table S5. Numerical values and confidence intervals for parameters obtained from experimental data of *L. brevis* predicted by equations (1-4) grown on alternative media formulated with thermal peptones (TP). R^2 is the determination coefficient among experimental and predicted data. NS: not significant. MRS was used as control commercial medium.

	**Sa_He**	**Sa_TF**	**Sa_Vis**	**RT_He**	**RT_TF**	**RT_Vis**	**Tu_He**	**Tu_TF**	**Tu_Vis**	**Sb_He**	**Sb_TF**	**Sb_Vis**	**Sbass_He**	**Sbass_TF**	**Sbass_Vis**	**MRS**
**Biomass (X)**																
X_m	4.76±0.41	4.85±0.23	4.77±0.26	4.52±0.48	4.74±0.29	4.23±0.27	4.17±0.16	4.53±0.39	4.17±0.30	4.30±0.21	4.30±0.16	4.29±0.44	4.38±0.18	4.39±0.49	4.46±0.32	4.58±0.11
v_m	0.57±0.17	0.44±0.08	0.51±0.12	0.55±0.16	0.58±0.10	0.42±0.11	0.36±0.10	0.47±0.14	0.32±0.08	0.48±0.12	0.44±0.08	0.35±0.13	0.48±0.12	0.25±0.07	0.33±0.08	0.54±0.09
λ_X	5.34±1.29	6.06±1.25	5.64±1.23	5.97±1.05	6.06±0.87	6.59±1.35	5.26±1.24	6.55±1.27	5.40±2.09	4.38±1.31	4.56±1.34	3.56±2.52	5.95±1.21	3.26±2.36	4.48±1.62	6.30±0.63
μ_X	0.48±0.19	0.36±0.14	0.43±0.11	0.49±0.13	0.49±0.10	0.40±0.11	0.35±0.12	0.42±0.11	0.31±0.13	0.44±0.15	0.41±0.11	0.32±0.14	0.44±0.12	0.23±0.08	0.30±0.08	0.47±0.09
τ_X	9.51±1.8	11.5±1.3	10.3±0.7	10.1±1.7	10.1±1.2	11.6±1.4	11.4±1.5	11.3±1.0	12.0±1.6	8.90±0.99	9.48±1.71	9.76±1.54	10.5±1.4	11.8±1.8	11.2±1.1	10.6±1.2
t_{mX}	13.7±3.6	17.1±1.6	15.0±1.6	14.2±3.4	14.2±2.6	16.7±2.6	16.8±2.5	16.1±2.0	18.5±1.9	13.4±2.5	14.4±1.8	16.0±3.5	15.1±1.3	20.4±1.7	17.9±2.5	14.5±1.5
R^2	0.989	0.997	0.995	0.990	0.997	0.995	0.983	0.987	0.993	0.991	0.993	0.983	0.991	0.994	0.993	0.996
**Cells (G)**																
G_m	12.2±1.3	12.7±1.0	12.0±1.1	12.4±1.2	12.5±1.5	12.3±1.3	12.3±1.0	12.2±1.5	12.0±1.8	12.5±1.4	12.4±1.4	12.4±1.3	12.1±1.7	11.8±2.0	11.9±1.1	12.3±1.3
v_G	0.95±0.28	1.85±0.39	1.19±0.49	1.24±0.35	1.02±0.29	0.92±0.33	1.12±0.38	1.12±0.41	1.34±0.46	1.24±0.50	0.96±0.38	1.00±0.39	0.91±0.46	1.43±0.37	1.11±0.43	1.54±0.65
λ_G	4.69±0.99	5.31±1.31	3.84±2.26	4.81±1.09	4.55±1.29	3.09±2.51	3.73±1.45	3.40±1.42	4.50±2.01	4.98±2.21	3.12±1.46	3.73±2.55	3.12(NS)	4.64±1.85	3.75±2.23	4.12±2.01
μ_G	0.31±0.11	0.58±0.15	0.39±0.17	0.40±0.11	0.33±0.10	0.30±0.12	0.47±0.15	0.59±0.16	0.60±0.20	0.40±0.10	0.31±0.09	0.32±0.14	0.30±0.17	0.48±0.14	0.37±0.16	0.50±0.10
τ_G	11.1±2.32	8.74±1.72	8.91±1.28	9.80±1.35	10.7±1.9	9.77±1.57	8.76±1.43	8.29±1.38	7.71±1.15	10.0±1.3	9.56±1.48	9.89±1.57	9.75±2.19	8.79±1.47	9.11±1.28	8.12±1.21
t_{mG}	17.5±3.0	12.1±2.8	14.0±2.9	14.8±2.5	16.8±2.8	16.4±3.6	13.0±2.1	11.7±1.6	11.1±1.8	15.0±1.9	16.0±2.7	16.1±3.6	16.4±5.0	12.9±2.7	14.5±2.9	12.1±2.5
R^2	0.981	0.953	0.983	0.997	0.994	0.984	0.992	0.984	0.979	0.987	0.984	0.983	0.967	0.988	0.985	0.977
**Lactic acid (L)**																
L_m	18.1±0.4	18.7±0.8	17.9±1.1	17.8±0.5	16.8±0.6	17.7±2.1	17.0±1.2	17.2±1.0	16.9±1.5	17.4±1.3	17.1±2.0	17.4±0.3	17.4±0.4	17.5±1.2	17.4±0.9	17.5±1.3
v_L	1.50±0.39	1.54±0.45	1.59±0.36	1.64±0.33	1.73±0.35	1.14±0.34	1.37±0.34	1.44±0.36	1.66±0.61	1.62±0.26	2.17±0.49	1.60±0.28	1.86±0.25	1.38±0.58	1.24±0.37	1.56±0.39
λ_L	5.76±0.48	5.93±0.69	5.94±1.33	6.10±0.55	6.04±0.49	4.74±2.42	5.64±3.71	5.72±3.02	5.97±1.95	4.81±1.92	6.12±1.01	4.91±1.32	5.48±1.12	4.36±3.04	4.85±1.46	5.94±0.72
μ_L	0.33±0.07	0.33±0.08	0.36±0.09	0.37±0.09	0.42±0.10	0.26±0.09	0.32±0.19	0.34±0.16	0.39±0.10	0.37±0.15	0.51±0.10	0.37±0.08	0.43±0.09	0.32±0.20	0.29±0.12	0.36±0.11
τ_L	11.8±0.6	12.0±0.9	11.6±0.9	11.5±0.3	10.9±0.6	12.5±1.9	11.9±0.9	11.7±1.2	11.0±0.8	10.2±1.8	10.1±1.6	10.4±1.4	10.2±0.8	10.7±2.2	11.9±2.1	11.5±1.5
t_{mL}	17.9±1.2	18.1±1.3	17.2±1.9	16.9±1.0	15.8±1.1	20.3±4.1	18.1±2.6	17.6±2.2	16.1±1.7	15.6±2.4	14.0±1.4	15.8±1.8	14.8±1.4	17.0±1.9	18.9±2.7	17.1±1.7
R^2	0.990	0.989	0.995	0.997	0.998	0.995	0.989	0.988	0.988	0.989	0.991	0.992	0.996	0.986	0.983	0.998
**Acetic acid (A)**																
A_m	1.29±0.26	1.79±0.26	2.06±0.49	1.97±0.16	2.13±0.18	1.89±0.54	1.56±0.24	1.62±0.19	1.70±0.35	2.10±0.50	2.10±0.72	1.96±0.22	1.80±0.28	2.14±0.48	2.31±0.48	1.87±0.40
v_A	0.08±0.03	0.15±0.06	0.12±0.05	0.11±0.04	0.13±0.05	0.11±0.06	0.14±0.07	0.17±0.07	0.20±0.06	0.12±0.05	0.10±0.06	0.11±0.02	0.12±0.06	0.12±0.05	0.11±0.02	0.12±0.05
λ_A	5.03±1.15	10.3±2.1	7.94±2.24	7.27±1.30	8.63±1.38	6.77±4.69	6.59±2.09	7.83±1.30	7.08±2.16	8.88±1.50	9.05±2.69	7.55±1.59	7.94±2.59	9.05±2.03	9.40±2.13	7.79±2.02
μ_A	0.24±0.11	0.34±0.11	0.23±0.09	0.23±0.10	0.24±0.10	0.24±0.16	0.35±0.12	0.43±0.10	0.46±0.18	0.22±0.09	0.20±0.14	0.22±0.05	0.27±0.11	0.23±0.12	0.20±0.07	0.26±0.09
τ_A	13.5±2.8	16.2±2.5	16.7±1.2	16.2±1.9	17.1±1.5	15.3±4.3	12.3±2.2	12.5±1.9	11.4±3.0	18.0±4.2	19.3±4.6	16.6±1.6	15.3±4.0	17.8±3.4	19.5±3.0	15.5±2.5
t_{mA}	22.0±3.1	22.0±2.9	25.4±7.9	25.0±2.6	25.5±2.0	23.7±9.2	18.0±1.6	17.2±4.1	15.8±3.0	27.2±7.8	29.5±9.8	25.7±3.5	22.6±3.3	26.6±5.3	29.6±5.9	23.3±2.9
R^2	0.965	0.993	0.990	0.988	0.991	0.952	0.976	0.990	0.987	0.996	0.990	0.995	0.987	0.992	0.991	0.983

Table S6. Maximum and minimum values of productive yields of LAB bioproductions. In brackets, the media that generated the corresponding yields are also indicated.

Yields (units)	*L. plantarum*	*L. brevis*
$Y_{X/RS}$ (gX/gRS)	0.119 (RT_He_FP)<br>0.180 (Sa_TF_FP)	0.171 (RT_Vis_FP)<br>0.243 (Sa_TF_FP)
$Y_{X/Pr}$ (gX/gPr)	1.13 (RT_TF_FP)<br>1.75 (Sa_TF_FP)	1.87 (Sb_TF_FP)<br>2.84 (RT_TF_FP)
$Y_{G/RS}$ (G/gRS)	0.427 (Tu_Vis_TP)<br>0.732 (Sa_TF_FP)	0.514 (Sbass_He_FP)<br>0.632 (Sa_He_TP)
$Y_{G/Pr}$ (G/gPr)	5.41 (Tu_Vis_TP)<br>8.91 (Sa_TF_FP)	5.02 (Sa_TF_FP)<br>7.28 (Sa_TF_TP)
$Y_{L/RS}$ (gL/gRS)	0.728 (RT_Vis_FP)<br>0.912 (MRS)	0.726 (Sbass_He_FP)<br>0.824 (Sb_TF_FP)
$Y_{L/Pr}$ (gL/gPr)	7.32 (RT_TF_FP)<br>11.1 (MRS)	6.79 (Sa_TF_FP)<br>9.25 (MRS)
$Y_{A/RS}$ (gA/gRS)	0.038 (Tu_He_TP)<br>0.084 (RT_TF_TP)	0.081 (Sb_TF_FP)<br>0.143 (RT_TF_FP)
$Y_{A/Pr}$ (gA/gPr)	0.452 (Tu_He_TP)<br>0.868 (Sa_He_FP)	0.78 (Sb_TF_FP)<br>1.74 (RT_TF_FP)

Table S7. Numerical values and confidence intervals for parameters obtained from experimental data of *Phaeobacter sp.* predicted by logistic equation grown on FP alternative media. R^2 is the determination coefficient among experimental and predicted data. The production yields ($Y_{X/Pr}$ and $Y_{G/Pr}$) are also shown. NS: not significant. MM was the control kinetic in commercial marine medium. Consistency of fittings were also determined (p-value from F-Fisher test).

Biomass (X)

	Sa_He	Sa_TF	Sa_Vis	RT_He	RT_TF	RT_Vis	Tu_He	Tu_TF	Tu_Vis	Sb_He	Sb_TF	Sb_Vis	Sbass_He	Sbass_TF	Sbass_Vis	MM
X_m	0.92±0.17	0.96±0.12	0.74±0.13	1.10±0.09	1.05±0.08	0.62±0.13	0.99±0.09	0.94±0.07	0.70±0.10	0.88±0.08	0.79±0.05	0.69±0.10	0.84±0.06	0.87±0.05	0.60±0.07	0.83±0.06
v_m	0.05±0.02	0.06±0.02	0.06±0.02	0.07±0.01	0.05±0.01	0.05±0.01	0.08±0.01	0.10±0.02	0.07±0.01	0.06±0.01	0.06±0.01	0.06±0.02	0.09±0.02	0.10±0.02	0.06±0.01	0.06±0.02
λ_X	4.01±3.19	4.80±2.31	6.81±2.53	6.57±1.45	5.15±1.18	5.77±1.21	4.88±1.36	5.06±0.87	5.14±0.69	5.04±1.27	5.48±1.03	5.17±1.41	5.39±0.96	7.20±1.10	7.65±1.09	2.27±2.01
μ_X	0.21±0.09	0.24±0.08	0.31±0.06	0.27±0.06	0.21±0.03	0.32±0.09	0.33±0.09	0.41±0.11	0.38±0.10	0.29±0.04	0.33±0.05	0.38±0.10	0.41±0.06	0.44±0.08	0.39±0.08	0.30±0.10
τ_X	13.5±2.9	13.1±1.9	13.2±1.2	14.0±1.2	14.9±1.2	12.0±1.4	10.9±1.0	9.92±0.96	10.4±1.09	12.0±1.0	11.6±1.2	10.7±1.2	10.3±1.0	11.8±1.3	12.8±1.4	8.91±1.21
t_{mX}	23.0±6.4	21.4±4.2	19.6±3.4	21.4±3.5	24.7±2.6	18.3±2.5	17.0±1.9	14.8±1.8	15.7±2.0	18.9±2.7	17.7±2.1	16.2±2.4	15.2±2.0	16.4±1.9	17.9±2.6	15.6±2.8
R^2	0.979	0.987	0.990	0.994	0.997	0.993	0.999	0.994	0.989	0.994	0.994	0.992	0.990	0.993	0.997	0.989
p-value	<0.0001	<0.0001	<0.0001	<0.0001	<0.0001	<0.0001	<0.0001	<0.0001	<0.0001	<0.0001	<0.0001	<0.0001	<0.0001	<0.0001	<0.0001	<0.0001
$Y_{X/Pr}$	0.506	0.486	0.465	0.627	0.539	0.448	0.586	0.503	0.472	0.539	0.473	0.441	0.468	0.535	0.410	0.834

Cells (G)

	Sa_He	Sa_TF	Sa_Vis	RT_He	RT_TF	RT_Vis	Tu_He	Tu_TF	Tu_Vis	Sb_He	Sb_TF	Sb_Vis	Sbass_He	Sbass_TF	Sbass_Vis	MM
G_m	13.7±2.2	14.1±2.1	13.4±1.8	13.8±1.3	13.6±1.6	12.2±1.2	13.4±1.4	13.5±1.4	12.4±0.8	13.7±1.7	13.2±1.5	12.1±1.3	13.4±1.4	13.0±1.5	12.8±1.1	13.6±1.2
v_G	0.95±0.50	1.04±0.54	1.11±0.39	1.09±0.38	1.07±0.47	1.28±0.35	1.07±0.34	1.41±0.39	1.14±0.28	0.89±0.35	1.07±0.39	1.27±0.31	0.78±0.32	0.92±0.29	1.01±0.39	1.12±0.36
λ_G	2.19(NS)	2.14(NS)	4.02(NS)	2.05(NS)	2.78(NS)	2.26±2.20	1.98(NS)	2.50(NS)	2.23±2.19	3.27(NS)	4.60(NS)	2.35(NS)	3.54(NS)	3.76(NS)	4.29(NS)	4.05±2.08
μ_G	0.28±0.17	0.30±0.17	0.32±0.14	0.31±0.12	0.32±0.16	0.42±0.10	0.32±0.10	0.42±0.11	0.37±0.09	0.26±0.19	0.33±0.15	0.42±0.11	0.23±0.09	0.28±0.13	0.32±0.14	0.33±0.12
τ_G	9.41±2.54	8.91±2.28	10.1±2.09	8.42±1.41	9.11±1.80	7.02±0.93	8.25±1.04	7.28±0.96	7.66±1.10	11.0±2.0	10.7±2.4	7.12±0.87	12.1±2.6	10.8±2.0	10.6±1.9	10.1±1.3
t_{mG}	16.6±5.9	15.7±5.3	16.1±3.9	14.8±3.2	15.5±4.1	11.8±4.0	14.5±2.3	12.1±2.1	13.1±2.6	18.6±3.6	16.9±4.1	11.9±3.7	20.7±4.6	17.9±5.3	16.9±3.9	16.1±2.9
R^2	0.962	0.965	0.994	0.984	0.977	0.988	0.970	0.990	0.990	0.965	0.986	0.991	0.982	0.980	0.970	0.987
p-value	<0.0001	<0.0001	<0.0001	<0.0001	<0.005	<0.0001	<0.0001	<0.0001	<0.0001	<0.0001	<0.0001	<0.0001	<0.0001	<0.0001	<0.0001	<0.0001
$Y_{G/Pr}$	7.78	7.74	7.02	8.33	7.55	9.41	8.08	7.83	8.23	8.81	7.49	8.37	7.22	8.07	9.74	12.96

Table S8. Numerical values and confidence intervals for parameters obtained from experimental data of *Phaeobacter sp.* predicted by logistic equation grown on TP alternative media. R^2 is the determination coefficient among experimental and predicted data. The production yields ($Y_{X/Pr}$ and $Y_{G/Pr}$) are also shown. NS: not significant. MM was the control kinetic in commercial marine medium. Consistency of fittings were also determined (p-value from F-Fisher test).

Biomass (X)

	Sa_He	Sa_TF	Sa_Vis	RT_He	RT_TF	RT_Vis	Tu_He	Tu_TF	Tu_Vis	Sb_He	Sb_TF	Sb_Vis	Sbass_He	Sbass_TF	Sbass_Vis	MM
X_m	0.96±0.13	0.95±0.12	0.94±0.09	1.03±0.12	0.99±0.10	0.85±0.12	0.91±0.08	1.00±0.12	0.82±0.09	0.93±0.10	0.86±0.11	0.75±0.10	0.90±0.10	0.90±0.09	0.73±0.10	0.87±0.12
v_m	0.07±0.03	0.05±0.02	0.07±0.03	0.07±0.02	0.05±0.01	0.06±0.02	0.07±0.02	0.07±0.01	0.08±0.02	0.09±0.03	0.07±0.03	0.06±0.02	0.07±0.02	0.07±0.02	0.06±0.01	0.07±0.02
λ_X	4.16±1.70	4.28±1.39	5.10±2.16	5.72±1.91	4.56±1.61	5.34±2.04	3.55±1.95	4.00±2.31	5.09±3.02	5.70±1.88	4.96±2.99	6.28±1.45	5.86±2.20	7.28±2.35	3.79(NS)	2.87±2.06
μ_X	0.28±0.09	0.21±0.06	0.29±0.10	0.26±0.08	0.21±0.05	0.28±0.07	0.31±0.10	0.30±0.07	0.40±0.11	0.37±0.08	0.32±0.10	0.32±0.08	0.29±0.09	0.33±0.09	0.32±0.07	0.33±0.09
τ_X	11.4±1.5	13.9±3.0	12.1±2.7	13.6±2.1	14.3±1.8	12.5±1.9	10.1±1.5	10.8±1.3	10.1±1.7	11.1±2.3	11.2±1.7	12.5±1.7	12.7±2.4	13.4±2.2	9.98±1.09	8.88±1.85
t_{mX}	18.6±2.6	23.6±3.2	19.1±2.0	21.4±2.5	24.0±3.5	19.6±3.3	16.6±2.8	17.5±2.6	15.2±6.4	16.5±3.4	17.4±3.0	18.8±2.8	19.6±2.0	19.5±3.6	16.2±2.7	14.9±3.0
R^2	0.981	0.989	0.992	0.995	0.994	0.988	0.996	0.990	0.993	0.993	0.976	0.993	0.994	0.989	0.986	0.993
p-value	<0.0001	<0.0001	<0.0001	<0.0001	<0.0001	<0.0001	<0.0001	<0.0001	<0.0001	<0.0001	<0.001	<0.0001	<0.0001	<0.0001	<0.0001	<0.0001
$Y_{X/Pr}$	0.495	0.506	0.503	0.512	0.501	0.487	0.499	0.524	0.475	0.510	0.467	0.452	0.498	0.505	0.420	0.738

Cells (G)

	Sa_He	Sa_TF	Sa_Vis	RT_He	RT_TF	RT_Vis	Tu_He	Tu_TF	Tu_Vis	Sb_He	Sb_TF	Sb_Vis	Sbass_He	Sbass_TF	Sbass_Vis	MM
G_m	13.8±1.7	13.3±1.4	13.9±1.4	13.9±1.5	13.5±1.6	13.5±1.7	13.1±1.5	13.5±1.0	13.3±1.9	13.4±1.7	13.2±1.2	12.9±1.6	13.3±1.3	13.1±1.3	12.5±1.8	13.6±1.4
v_G	0.96±0.29	0.97±0.24	0.97±0.32	1.07±0.35	1.11±0.30	1.10±0.28	1.10±0.32	1.17±0.19	0.91±0.27	0.91±0.28	0.90±0.25	0.91±0.26	0.80±0.30	0.83±0.25	1.06±0.29	1.02±0.28
λ_G	2.93(NS)	2.39(NS)	1.99(NS)	2.12(NS)	2.52(NS)	3.01±2.86	2.11(NS)	2.88(NS)	2.16(NS)	3.52(NS)	4.02(NS)	4.78±2.15	4.56±3.61	4.05(NS)	4.80±2.20	2.97(NS)
μ_G	0.28(NS)	0.29(NS)	0.28(NS)	0.31(NS)	0.33(NS)	0.33(NS)	0.34(NS)	0.35±0.20	0.28(NS)	0.27(NS)	0.27(NS)	0.28±0.16	0.24±0.19	0.25(NS)	0.34±0.20	0.30±0.23
τ_G	10.1±2.01	9.19±1.85	9.13±2.21	8.64±2.15	8.60±1.85	9.12±1.99	8.08±1.79	8.67±1.34	9.45±2.21	10.9±1.9	11.3±2.4	11.9±2.7	12.8±3.3	11.9±2.1	10.7±2.1	9.59±1.61
t_{mG}	17.3±2.1	16.0±3.2	16.3±2.8	15.2±2.8	14.7±2.5	15.2±3.6	14.0±2.2	14.5±2.8	16.7±3.0	18.2±2.8	18.7±3.0	19.0±3.0	21.1±5.0	19.8±3.3	16.7±2.6	13.2±3.0
R^2	0.983	0.981	0.970	0.977	0.981	0.990	0.984	0.991	0.980	0.974	0.986	0.992	0.986	0.984	0.991	0.987
p-value	<0.001	<0.0001	<0.0001	<0.001	<0.0001	<0.0001	<0.0001	<0.0001	<0.0001	<0.001	<0.0001	<0.0001	<0.0001	<0.0001	<0.0001	<0.0001
$Y_{G/Pr}$	7.89	7.82	7.89	7.95	7.82	7.85	7.70	7.76	7.69	7.55	7.60	7.35	7.59	7.63	7.15	10.91

Table S9. Numerical values and confidence intervals for parameters obtained from experimental data of *P. fluorescens* predicted by logistic equation grown on FP alternative media. R^2 is the determination coefficient among experimental and predicted data. The production yields ($Y_{X/Pr}$ and $Y_{G/Pr}$) are also shown. NS: not significant. MM was the control kinetic in commercial marine medium. Consistency of fittings were also determined (p-value from F-Fisher test).

Biomass (X)

	Sa_He	Sa_TF	Sa_Vis	RT_He	RT_TF	RT_Vis	Tu_He	Tu_TF	Tu_Vis	Sb_He	Sb_TF	Sb_Vis	Sbass_He	Sbass_TF	Sbass_Vis	MM
X_m	0.86±0.31	1.03±0.54	0.63±0.16	0.71±0.12	0.88±0.31	0.78±0.49	0.86±0.29	0.88±0.30	0.77±0.27	0.87±0.39	0.96±0.61	0.89±0.29	0.84±0.39	0.90±0.72	0.84±0.51	0.91±0.23
v_m	0.04±0.01	0.03±0.01	0.06±0.03	0.07±0.06	0.03±0.01	0.05±0.04	0.04±0.03	0.04±0.03	0.05±0.04	0.04±0.03	0.04±0.03	0.04±0.03	0.04±0.03	0.03±0.02	0.04±0.03	0.07±0.04
λ_X	10.7±2.7	1.23(NS)	3.85±2.16	1.58(NS)	4.69±3.60	1.91(NS)	1.27(NS)	1.48(NS)	0.66(NS)	0.72(NS)	1.42(NS)	1.87(NS)	0.68(NS)	0.76(NS)	0.97(NS)	1.52(NS)
μ_X	0.18±0.08	0.13±0.08	0.39±0.14	0.40±0.34	0.15±0.07	0.25(NS)	0.18(NS)	0.18(NS)	0.23(NS)	0.18(NS)	0.16(NS)	0.18(NS)	0.21(NS)	0.15(NS)	0.18(NS)	0.29(NS)
τ_X	21.8±5.1	17.2±10.5	8.99±2.71	6.64±2.46	17.9±6.1	9.89±5.00	12.2±3.9	12.8±5.1	9.23±3.61	12.0±9.2	14.4±10.7	12.9±4.4	10.3±8.0	14.1±9.9	12.0±10.3	8.44±3.33
t_{mX}	33.0±9.5	33.1±20.4	14.1±4.6	11.7±5.4	31.2±12.0	17.9±5.3	23.1±8.0	24.1±6.7	17.8±5.4	23.2±18.1	27.3±17.6	23.9±6.5	19.9±9.3	27.5±13.2	23.8±15.2	15.4±7.9
R^2	0.984	0.953	0.978	0.926	0.976	0.986	0.981	0.980	0.961	0.976	0.973	0.977	0.963	0.962	0.977	0.987
p-value	<0.0001	<0.001	<0.0001	<0.001	<0.0001	<0.0001	<0.0001	<0.0001	<0.0001	<0.0001	<0.0001	<0.0001	<0.0001	<0.0001	<0.0001	<0.0001
$Y_{X/Pr}$	0.347	0.404	0.375	0.544	0.409	0.383	0.402	0.410	0.397	0.571	0.563	0.407	0.541	0.520	0.540	0.842

Cells (G)

	Sa_He	Sa_TF	Sa_Vis	RT_He	RT_TF	RT_Vis	Tu_He	Tu_TF	Tu_Vis	Sb_He	Sb_TF	Sb_Vis	Sbass_He	Sbass_TF	Sbass_Vis	MM
G_m	14.8±3.5	14.6±2.3	13.6±2.0	14.8±4.5	15.2±2.1	13.6±1.9	14.3±1.9	14.0±1.7	13.9±1.6	13.6±2.0	13.1±2.5	13.8±2.0	13.0±2.2	13.5±2.3	13.6±1.8	15.3±2.4
v_G	0.93±0.27	1.24±0.79	1.03±0.41	0.87±0.78	1.06±0.49	1.10±0.61	1.49±0.49	1.24±0.55	1.15±0.42	0.85±0.51	1.19±0.62	1.10±0.45	0.90±0.56	0.90±0.54	0.78±0.60	1.48±0.92
λ_G	1.57(NS)	2.41(NS)	3.29±2.41	0.58(NS)	2.44(NS)	2.94(NS)	3.86(NS)	2.93(NS)	3.83(NS)	3.65(NS)	5.23(NS)	2.51(NS)	3.27(NS)	4.35(NS)	4.61(NS)	1.95 (NS)
μ_G	0.25±0.21	0.34±0.24	0.30±0.15	0.24(NS)	0.28±0.15	0.32±0.20	0.42±0.29	0.36±0.30	0.33±0.28	0.25±0.20	0.36±0.14	0.32±0.27	0.28±0.21	0.27±0.19	0.23±0.21	0.39±0.26
τ_G	9.54±3.88	8.28±2.34	9.91±1.92	9.07±5.12	9.60±2.23	9.16±2.78	8.65±3.16	8.57±3.09	9.87±4.10	11.7±3.7	10.7±3.8	8.79±3.67	10.5±4.0	11.7±3.5	13.3±5.0	7.14±1.97
t_{mG}	17.5±9.1	14.2±5.3	16.5±3.8	17.6±1.2	16.8±5.1	15.4±3.0	13.4±3.2	14.2±2.8	15.9±3.9	19.7±6.4	16-2±5.9	15.1±2.7	17.7±4.2	19.4±4.8	22.1±7.4	12.3±4.3
R^2	0.924	0.953	0.988	0.882	0.971	0.987	0.995	0.981	0.983	0.985	0.973	0.981	0.982	0.988	0.979	0.956
p-value	<0.0001	<0.0001	<0.0001	<0.005	<0.0001	<0.0001	<0.0001	<0.0001	<0.0001	<0.0001	<0.0001	<0.0001	<0.0001	<0.0001	<0.0001	<0.0001
$Y_{G/Pr}$	7.17	7.10	6.92	9.65	7.96	9.85	8.40	8.62	7.98	9.44	8.85	9.17	8.57	8.59	9.11	14.03

Table S10. Numerical values and confidence intervals for parameters obtained from experimental data of *P. fluorescens* predicted by logistic equation grown on TP alternative media. R^2 is the determination coefficient among experimental and predicted data. The production yields ($Y_{X/Pr}$ and $Y_{G/Pr}$) are also shown. NS: not significant. MM was the control kinetic in commercial marine medium. Consistency of fittings were also determined (p-value from F-Fisher test).

Biomass (X)

	Sa_He	Sa_TF	Sa_Vis	RT_He	RT_TF	RT_Vis	Tu_He	Tu_TF	Tu_Vis	Sb_He	Sb_TF	Sb_Vis	Sbass_He	Sbass_TF	Sbass_Vis	MM
X_m	0.82±0.34	0.77±0.41	0.72±0.08	0.77±0.19	0.81±0.28	0.74±0.08	0.80±0.26	0.77±0.32	0.74±0.36	0.82±0.18	0.81±0.26	0.76±0.30	0.79±0.16	0.83±0.19	0.73±0.32	0.85±0.10
v_m	0.04±0.02	0.05±0.04	0.05±0.02	0.04(NS)	0.04±0.03	0.06±0.02	0.04±0.03	0.04±0.03	0.04±0.03	0.05±0.02	0.05±0.03	0.05±0.03	0.04±0.01	0.05±0.02	0.04±0.03	0.06±0.03
λ_X	7.35±3.12	2.22(NS)	1.41(NS)	1.22(NS)	5.30±4.10	2.51(NS)	0.75(NS)	2.43(NS)	0.57(NS)	2.10(NS)	1.51(NS)	2.20(NS)	2.25(NS)	4.11(NS)	0.20(NS)	0.77(NS)
μ_X	0.20±0.12	0.24(NS)	0.29±0.13	0.20(NS)	0.20±0.11	0.32±0.13	0.20(NS)	0.20(NS)	0.21(NS)	0.23(NS)	0.23(NS)	0.27(NS)	0.22±0.15	0.23±0.17	0.22(NS)	0.30±0.15
τ_X	17.6±4.9	10.6±6.6	8.38±1.83	11.1±4.9	15.3±5.1	9.27±1.44	10.7±6.0	12.6±6.7	10.0±5.8	10.7±3.8	10.3±2.9	9.76±4.15	11.2±2.7	12.8±3.6	9.17±6.2	7.5±1.9
t_{mX}	27.8±10.3	19.0±8.2	15.3±4.2	21.0±9.6	25.4±10.8	15.4±3.3	20.7±7.0	22.8±6.7	19.5±8.2	19.4±5.0	19.2±5.5	17.3±7.9	20.3±3.6	21.5±4.1	18.1±10.3	14.3±4.1
R^2	0.990	0.958	0.975	0.965	0.976	0.983	0.972	0.980	0.971	0.976	0.984	0.987	0.989	0.983	0.976	0.970
p-value	<0.0001	<0.001	<0.0001	<0.001	<0.0001	<0.0001	<0.001	<0.0001	<0.001	<0.001	<0.0001	<0.001	<0.001	<0.001	<0.001	<0.001
$Y_{X/Pr}$	0.461	0.357	0.341	0.360	0.480	0.348	0.369	0.367	0.353	0.429	0.415	0.359	0.365	0.432	0.401	0.789

Cells (G)

	Sa_He	Sa_TF	Sa_Vis	RT_He	RT_TF	RT_Vis	Tu_He	Tu_TF	Tu_Vis	Sb_He	Sb_TF	Sb_Vis	Sbass_He	Sbass_TF	Sbass_Vis	MM
G_m	14.2±3.8	14.4±3.7	13.7±2.9	14.6±4.0	14.3±3.6	14.0±2.6	14.4±2.1	14.0±3.5	14.3±2.6	13.6±2.2	13.5±3.0	13.5±1.8	14.1±2.4	13.8±2.3	13.4±2.3	14.8±3.1
v_G	1.02±0.60	0.99±0.58	0.93±0.40	0.92±0.63	1.15±0.51	0.96±0.40	0.83±0.26	1.07±0.34	0.86±0.31	0.85±0.16	0.98±0.29	0.80±0.24	0.85±0.32	1.16±0.34	0.72±0.26	1.38±0.60
λ_G	3.10(NS)	2.30(NS)	2.67(NS)	2.33(NS)	3.05(NS)	2.95(NS)	2.47(NS)	2.93(NS)	3.14(NS)	3.65(NS)	4.60±2.81	4.33±2.59	4.02(NS)	5.44±3.94	2.69(NS)	2.42(NS)
μ_G	0.29±0.20	0.28±0.19	0.27±0.20	0.25±0.21	0.32±0.16	0.27(NS)	0.23±0.10	0.31(NS)	0.24±0.13	0.25±0.21	0.29±0.09	0.24±0.09	0.24(NS)	0.34±0.09	0.21±0.10	0.38±0.17
τ_G	10.0±3.9	9.57±2.8	10.0±2.2	10.3±4.6	9.30±3.33	10.3±2.8	11.1±2.0	9.49±3.36	11.4±2.8	11.4±2.5	11.5±2.1	12.8±2.1	12.3±3.3	11.4±1.8	12.0±2.8	7.80±2.60
t_{mG}	17.0±6.2	16.8±5.6	17.4±4.8	18.2±9.0	15.6±5.7	17.6±3.8	19.8±3.0	16.0±4.2	17.6±3.8	19.5±1.9	18.4±2.3	21.2±4.7	20.6±3.0	17.3±2.7	21.4±6.4	13.2±5.4
R^2	0.975	0.967	0.976	0.963	0.977	0.983	0.974	0.979	0.983	0.983	0.981	0.984	0.988	0.981	0.975	0.976
p-value	<0.001	<0.001	<0.0001	<0.0001	<0.001	<0.0001	<0.0001	<0.0001	<0.0001	<0.0001	<0.0001	<0.0001	<0.0001	<0.0001	<0.001	<0.001
$Y_{G/Pr}$	8.32	7.99	7.52	8.93	9.21	9.06	8.52	8.19	7.98	7.90	8.23	8.48	8.90	8.29	8.05	12.95

Table S11. Numerical values and confidence intervals for parameters obtained from experimental data of *B. subtilis* predicted by logistic equation grown on FP alternative media. R^2 is the determination coefficient among experimental and predicted data. The production yields ($Y_{X/RS}$, $Y_{X/Pr}$, $Y_{G/RS}$ and $Y_{G/Pr}$) are also shown. NS: not significant. TSB was the control kinetic in commercial TSB medium. Consistency of fittings were also determined (p-value from F-Fisher test).

Biomass (X)

	**Sa_He**	**Sa_TF**	**Sa_Vis**	**RT_He**	**RT_TF**	**RT_Vis**	**Tu_He**	**Tu_TF**	**Tu_Vis**	**Sb_He**	**Sb_TF**	**Sb_Vis**	**Sbass_He**	**Sbass_TF**	**Sbass_Vis**	**TSB**
X_m	0.94±0.12	0.99±0.08	0.78±0.11	1.11±0.10	1.11±0.09	0.81±0.11	0.95±0.09	1.03±0.10	0.87±0.09	0.91±0.10	0.99±0.08	0.94±0.09	0.93±0.09	0.88±0.08	0.82±0.06	1.16±0.11
v_m	0.08±0.04	0.07±0.01	0.10±0.03	0.07±0.02	0.08±0.02	0.07±0.02	0.07±0.02	0.08±0.02	0.06±0.01	0.07±0.01	0.07±0.02	0.06±0.01	0.07±0.01	0.05±0.01	0.07±0.03	0.08±0.02
λ_X	0.08 (NS)	4.22±1.62	6.07±2.10	3.36±1.96	3.27±1.85	3.99±1.76	2.66±1.61	2.97±2.34	2.50±2.16	2.58±2.07	2.92±2.71	3.24±2.19	2.82±2.63	0.96(NS)	3.85±1.60	2.01(NS)
μ_X	0.32±0.19	0.27±0.07	0.52±0.09	0.25±0.07	0.29±0.09	0.35±0.10	0.30±0.08	0.30±0.06	0.29±0.07	0.30±0.07	0.29±0.08	0.27±0.09	0.29±0.07	0.23±0.06	0.34±0.07	0.26±0.09
τ_X	7.04±2.02	11.7±1.2	9.94±1.99	11.5±1.4	10.1±1.2	9.66±1.81	9.31±1.56	9.75±1.71	9.39±2.03	9.35±1.43	9.81±1.50	10.6±2.2	9.67±1.36	9.85±1.61	9.50±1.46	9.62±1.58
t_{mX}	13.3±4.6	19.1±2.6	13.8±3.1	19.6±3.3	17.0±2.7	15.3±2.4	16.0±2.8	16.5±3.6	16.3±3.2	16.1±2.9	16.7±3.1	18.0±3.0	16.5±3.4	18.7±3.8	15.5±2.2	17.2±3.7
R^2	0.961	0.994	0.998	0.992	0.992	0.996	0.988	0.991	0.994	0.992	0.993	0.955	0.997	0.976	0.981	0.986
p-value	<0.005	<0.0001	<0.0001	<0.0001	<0.0001	<0.0001	<0.0001	<0.0001	<0.0001	<0.0001	<0.0001	<0.005	<0.0001	<0.0001	<0.0001	<0.0001
$Y_{X/RS}$	0.351	0.373	0.330	0.434	0.431	0.264	0.377	0.383	0.301	0.358	0.413	0.337	0.371	0.356	0.358	0.494
$Y_{X/Pr}$	0.552	0.614	0.503	0.581	0.483	0.506	0.589	0.660	0.523	0.438	0.601	0.514	0.481	0.512	0.466	0.658

Cells (G)

	**Sa_He**	**Sa_TF**	**Sa_Vis**	**RT_He**	**RT_TF**	**RT_Vis**	**Tu_He**	**Tu_TF**	**Tu_Vis**	**Sb_He**	**Sb_TF**	**Sb_Vis**	**Sbass_He**	**Sbass_TF**	**Sbass_Vis**	**TSB**
G_m	7.40±0.73	7.09±0.55	7.63±1.51	7.37±0.47	7.43±0.32	7.77±1.01	7.98±1.13	8.31±0.89	8.04±1.09	8.30±0.93	8.35±1.00	7.74±1.17	8.11±0.78	8.02±1.16	7.86±0.99	8.41±1.00
v_G	0.92±0.48	2.16 (NS)	0.93±0.37	2.11±1.99	1.66±0.60	0.67±0.40	0.72±0.53	0.61±0.39	0.75±0.48	1.04±0.47	0.56±0.38	0.92±0.56	0.93±0.53	0.69±0.40	0.79±0.37	0.88±0.50
λ_G	3.97±2.35	4.52±2.17	4.33±1.81	4.55±1.50	4.43±0.83	2.46±1.80	2.74±2.31	2.19±2.07	2.63±2.30	4.10±1.21	2.23(NS)	3.31±2.85	3.95±1.39	4.47(NS)	2.99(NS)	3.09±3.00
μ_G	0.50±0.27	1.22 (NS)	0.49±0.09	1.15±1.10	0.89±0.34	0.35±0.28	0.36±0.19	0.29±0.16	0.37±0.20	0.50±0.29	0.27±0.22	0.48±0.21	0.46±0.25	0.34±0.26	0.40±0.21	0.42±0.25
τ_G	7.99±1.28	6.16±0.64	8.42±0.91	6.29±0.58	6.67±0.47	8.27±2.22	8.33±1.52	9.05±1.37	8.03±1.49	8.07±1.40	9.76±1.96	7.50±1.80	8.33±2.40	10.3±2.8	7.97±2.09	7.88±1.67
t_{mG}	12.0±2.7	7.80±2.65	12.5±2.0	8.03±2.01	8.91±1.08	14.1±3.6	13.9±2.8	15.9±3.3	13.4±2.9	12.0±3.0	17.3±5.4	11.7±3.1	12.7±3.8	16.1±4.7	13.0±3.4	12.7±3.64
R^2	0.976	0.980	0.992	0.987	0.994	0.977	0.965	0.955	0.977	0.961	0.953	0.977	0.975	0.944	0.963	0.968
p-value	<0.0001	<0.0001	<0.0001	<0.0001	<0.0001	<0.0001	<0.0001	<0.005	<0.0001	<0.005	<0.005	<0.0001	<0.0001	<0.005	<0.005	<0.005
$Y_{G/RS}$	3.95	3.82	3.89	3.59	2.99	5.54	5.13	5.51	4.67	3.76	5.22	4.35	4.39	4.98	4.21	4.21
$Y_{X/Pr}$	2.52	2.30	2.37	2.69	2.65	2.89	3.29	3.20	2.68	3.08	3.55	2.86	3.34	3.46	3.24	3.24

Table S12. Numerical values and confidence intervals for parameters obtained from experimental data of *B. subtilis* predicted by logistic equation grown on TP alternative media. R^2 is the determination coefficient among experimental and predicted data. The production yields ($Y_{X/RS}$, $Y_{X/Pr}$, $Y_{G/RS}$ and $Y_{G/Pr}$) are also shown. NS: not significant. TSB was the control kinetic in commercial TSB medium. Consistency of fittings were also determined (p-value from F-Fisher test).

Biomass (X)

	Sa_He	Sa_TF	Sa_Vis	RT_He	RT_TF	RT_Vis	Tu_He	Tu_TF	Tu_Vis	Sb_He	Sb_TF	Sb_Vis	Sbass_He	Sbass_TF	Sbass_Vis	TSB
X_m	1.03±0.23	1.00±0.09	0.97±0.13	1.01±0.10	0.99±0.25	0.91±0.10	0.91±0.12	0.92±0.14	0.92±0.16	0.95±0.09	0.91±0.11	0.93±0.10	1.02±0.11	1.09±0.13	0.96±0.15	1.15±0.26
v_m	0.07±0.04	0.08±0.03	0.05±0.04	0.09±0.02	0.05±0.03	0.09±0.03	0.06±0.02	0.08±0.04	0.05±0.02	0.07±0.02	0.06±0.02	0.06±0.02	0.05±0.03	0.05±0.02	0.07±0.04	0.07±0.04
λ_X	2.26±2.01	4.15±1.61	0.95(NS)	3.15±1.61	2.19(NS)	2.12±2.00	2.36±2.20	4.20±2.81	2.49(NS)	2.75±2.10	1.92±1.80	2.34(NS)	1.56(NS)	3.23(NS)	1.77(NS)	1.04(NS)
μ_X	0.29±0.20	0.33±0.13	0.22±0.19	0.37±0.07	0.22±0.17	0.39±0.19	0.25±0.17	0.33±0.20	0.20±0.09	0.29±0.14	0.26±0.15	0.27±0.12	0.25±0.17	0.19(NS)	0.29±0.18	0.24(NS)
τ_X	9.20±2.40	10.3±1.0	10.1±2.9	8.60±1.53	11.3±2.3	7.29±2.16	10.5±1.8	10.3±1.6	12.5±3.0	9.59±1.73	9.71±1.84	9.65±1.68	9.72±1.90	14.0±8.6	8.60±2.41	9.28±3.06
t_{mX}	16.1±2.6	16.5±2.0	19.3±3.8	14.0±1.6	20.5±2.9	12.4±1.9	18.6±4.2	16.4±2.5	22.5±6.7	16.4±4.2	17.5±3.7	17.0±3.0	17.9±3.9	24.7±13.6	15.4±5.6	17.5±4.1
R^2	0.964	0.992	0.969	0.992	0.980	0.981	0.986	0.972	0.978	0.993	0.991	0.993	0.984	0.986	0.957	0.966
p-value	<0.001	<0.0001	<0.001	<0.0001	<0.0001	<0.0001	<0.0001	<0.001	<0.001	<0.0001	<0.001	<0.0001	<0.0001	<0.0001	<0.005	<0.001
$Y_{X/RS}$	0.372	0.363	0.371	0.392	0.351	0.333	0.320	0.301	0.330	0.353	0.332	0.343	0.380	0.402	0.335	0.423
$Y_{X/Pr}$	0.591	0.582	0.570	0.563	0.544	0.514	0.531	0.502	0.522	0.561	0.554	0.521	0.581	0.593	0.551	0.611

Cells (G)

	Sa_He	Sa_TF	Sa_Vis	RT_He	RT_TF	RT_Vis	Tu_He	Tu_TF	Tu_Vis	Sb_He	Sb_TF	Sb_Vis	Sbass_He	Sbass_TF	Sbass_Vis	TSB
G_m	7.59±0.61	7.28±0.59	7.46±0.83	7.65±0.48	7.58±0.45	7.49±0.72	7.43±0.51	7.59±0.70	7.55±0.60	7.69±1.95	7.94±2.06	7.60±1.28	7.89±1.84	7.82±1.24	7.58±0.93	7.98±0.60
v_G	0.73±0.28	1.15±0.40	0.66±0.31	1.21±0.49	1.00±0.39	0.62±0.24	0.57±0.26	0.75±0.30	0.77±0.29	0.85±0.41	0.50±0.31	0.66±0.28	0.57±0.40	0.54±0.21	0.55±0.23	1.20±0.53
λ_G	3.22±1.85	3.37(NS)	2.60(NS)	3.93±3.02	3.78(NS)	2.78±2.47	2.97±1.52	3.15±2.85	2.86±2.10	3.85±3.01	2.48(NS)	3.63±2.70	2.78(NS)	3.73±2.84	2.75(NS)	3.57(NS)
μ_G	0.39±0.16	0.63±0.28	0.35±0.18	0.63±0.30	0.53±0.25	0.33±0.14	0.31±0.12	0.40±0.15	0.41±0.13	0.44±0.19	0.25(NS)	0.35±0.17	0.29(NS)	0.28±0.21	0.29±0.14	0.64±0.27
τ_G	8.42±2.01	6.54±1.87	8.27±1.65	7.10±2.21	7.56±1.90	8.82±1.45	9.44±1.70	8.20±1.16	7.77±1.81	8.40±2.71	10.5±2.2	9.40±1.94	9.68±2.24	11.0±2.8	9.59±1.92	6.69±2.21
t_{mG}	13.6±2.4	9.70±3.12	14.0±3.7	10.3±2.0	11.3±3.1	14.9±3.3	15.9±3.0	13.3±2.4	12.7±3.9	13.0±1.4	18.5±3.1	15.2±2.3	16.6±2.9	18.3±3.6	16.4±4.4	9.80±4.24
R^2	0.992	0.996	0.975	0.995	0.993	0.982	0.982	0.975	0.972	0.974	0.973	0.987	0.972	0.982	0.978	0.993
p-value	<0.0001	<0.0001	<0.001	<0.0001	<0.0001	<0.0001	<0.0001	<0.001	<0.001	<0.001	<0.001	<0.0001	<0.001	<0.0001	<0.0001	<0.0001
$Y_{G/RS}$	3.87	3.70	3.95	3.81	3.69	4.02	3.85	3.76	3.76	3.90	4.10	3.74	3.99	4.01	3.80	4.15
$Y_{X/Pr}$	2.51	2.44	2.58	2.55	2.52	2.71	2.60	2.52	2.58	2.68	2.75	2.60	2.64	2.72	2.62	3.05

Table S13. Numerical values and confidence intervals for parameters obtained from experimental data of *S. epidermidis* predicted by logistic equation grown on FP alternative media. R^2 is the determination coefficient among experimental and predicted data. The production yields ($Y_{X/RS}$, $Y_{X/Pr}$, $Y_{G/RS}$ and $Y_{G/Pr}$) are also shown. NS: not significant. TSB was the control kinetic in commercial TSB medium. Consistency of fittings were also determined (p-value from F-Fisher test).

Biomass (X)

	**Sa_He**	**Sa_TF**	**Sa_Vis**	**RT_He**	**RT_TF**	**RT_Vis**	**Tu_He**	**Tu_TF**	**Tu_Vis**	**Sb_He**	**Sb_TF**	**Sb_Vis**	**Sbass_He**	**Sbass_TF**	**Sbass_Vis**	**TSB**
X_m	2.18±0.15	1.87±0.07	1.62±0.12	1.79±0.13	1.51±0.17	1.62±0.11	1.83±0.14	1.91±0.12	1.75±0.11	2.00±0.12	1.88±0.15	1.75±0.12	1.78±0.18	1.71±0.16	1.80±0.19	2.60±0.16
v_m	0.32±0.13	0.23±0.05	0.13±0.07	0.17±0.06	0.16±0.10	0.17±0.03	0.18±0.04	0.17±0.06	0.17±0.05	0.20±0.09	0.19±0.08	0.20±0.04	0.17±0.10	0.20±0.11	0.18±0.07	0.32±0.11
λ_X	3.29±1.58	3.84±0.95	0.97(NS)	0.72(NS)	-0.64(NS)	3.11±1.79	3.13±2.24	1.57(NS)	2.60±2.09	2.26±1.81	2.42±1.69	2.98±2.61	2.56±2.01	2.03±1.62	1.95±1.34	1.47(NS)
μ_X	0.59±0.25	0.49±0.11	0.33±0.08	0.39±0.14	0.43±0.28	0.41±0.10	0.39±0.13	0.35±0.14	0.39±0.15	0.40±0.17	0.39±0.19	0.46±0.10	0.38±0.20	0.48±0.16	0.40±0.13	0.49±0.18
τ_X	6.71±0.85	7.93±0.52	7.10±1.01	5.84±1.06	4.05±1.68	7.93±1.48	8.33±1.09	7.35±1.19	7.74±1.43	7.29±1.01	7.50±1.12	7.31±1.50	7.84±1.26	6.24±1.20	6.99±1.09	5.58±0.87
t_{mX}	10.1±1.8	12.0±1.1	13.2±1.7	11.0±2.3	8.74±3.73	12.8±2.3	13.5±2.0	13.1±1.9	12.9±2.0	12.3±1.9	12.6±2.0	11.6±1.8	13.1±2.1	10.4±2.2	12.0±2.4	9.68±1.87
R^2	0.987	0.996	0.960	0.985	0.948	0.976	0.998	0.984	0.997	0.989	0.989	0.998	0.997	0.992	0.990	0.987
p-value	<0.0001	<0.0001	<0.001	<0.0001	<0.005	<0.0001	<0.0001	<0.0001	<0.0001	<0.0001	<0.0001	<0.0001	<0.0001	<0.0001	<0.0001	<0.0001
$Y_{X/RS}$	0.731	0.653	0.642	0.682	0.581	0.782	0.821	0.863	0.653	0.970	0.885	0.791	0.680	0.797	0.862	0.972
$Y_{X/Pr}$	0.891	1.16	1.02	0.924	0.720	0.976	1.02	1.03	0.899	1.204	1.05	0.852	0.936	0.858	1.069	1.38

Cells (G)

	**Sa_He**	**Sa_TF**	**Sa_Vis**	**RT_He**	**RT_TF**	**RT_Vis**	**Tu_He**	**Tu_TF**	**Tu_Vis**	**Sb_He**	**Sb_TF**	**Sb_Vis**	**Sbass_He**	**Sbass_TF**	**Sbass_Vis**	**TSB**
G_m	7.49±1.16	7.36±0.69	7.19±0.97	6.72±0.73	7.24±0.41	7.06±2.92	7.58±1.16	7.31±2.69	7.13±2.01	7.36±1.81	7.15±2.16	6.80±2.30	7.47±2.09	7.46±1.92	7.42±1.97	7.87±1.30
v_G	1.11(NS)	2.94(NS)	0.88±0.43	1.09±0.79	1.49±0.71	0.79±0.51	0.44±0.19	0.59±0.30	0.61±0.28	0.61(NS)	0.83±0.64	0.57±0.24	0.67±0.49	0.41(NS)	0.71±0.63	0.61±0.39
λ_G	3.86±3.85	5.05±3.22	3.22±2.65	3.31±2.50	3.98±1.20	0.49(NS)	-0.09(NS)	0.06(NS)	0.14(NS)	0.96(NS)	2.79±2.61	-0.35(NS)	1.23(NS)	-0.91(NS)	1.43(NS)	1.83(NS)
μ_G	0.69(NS)	1.60(NS)	0.49±0.38	0.65±0.49	0.83±0.40	0.45±0.43	0.23±0.16	0.32±0.21	0.34±0.20	0.33±0.24	0.47±0.30	0.34±0.22	0.36±0.21	0.22±0.19	0.39(NS)	0.31±0.22
τ_G	6.78±2.10	6.31±1.19	7.32±1.56	6.40±1.31	6.40±0.60	4.93±4.16	8.53±3.60	6.28±3.76	6.01±4.06	7.00±3.05	7.08±2.74	5.60±3.88	6.80±3.15	8.12±3.42	6.63±2.81	8.24±2.56
t_{mG}	9.70±4.49	7.56±5.29	11.4±4.6	9.49±2.86	8.83±1.44	9.38±9.21	17.1±6.9	12.5±8.9	11.9±8.7	13.0±6.5	11.4±5.9	11.6±8.2	12.4±7.0	17.1±7.5	11.8±6.1	14.7±5.9
R^2	0.949	0.973	0.994	0.962	0.990	0.939	0.972	0.939	0.947	0.976	0.954	0.949	0.926	0.930	0.952	0.949
p-value	<0.0001	<0.0001	<0.0001	<0.0001	<0.0001	<0.005	<0.0001	<0.005	<0.005	<0.0001	<0.005	<0.005	<0.005	<0.005	<0.005	<0.005
$Y_{G/RS}$	3.40	5.57	4.65	3.85	3.56	4.45	4.38	4.16	4.26	4.39	3.92	3.55	4.33	3.65	4.27	4.81
$Y_{X/Pr}$	2.78	3.11	2.93	2.82	2.87	3.57	3.53	3.50	3.10	3.54	3.30	3.30	3.14	3.39	3.44	3.39

Table S14. Numerical values and confidence intervals for parameters obtained from experimental data of *S. epidermidis* predicted by logistic equation grown on TP alternative media. R^2 is the determination coefficient among experimental and predicted data. The production yields ($Y_{X/RS}$, $Y_{X/Pr}$, $Y_{G/RS}$ and $Y_{G/Pr}$) are also shown. NS: not significant. TSB was the control kinetic in commercial TSB medium. Consistency of fittings were also determined (p-value from F-Fisher test).

Biomass (X)

	Sa_He	Sa_TF	Sa_Vis	RT_He	RT_TF	RT_Vis	Tu_He	Tu_TF	Tu_Vis	Sb_He	Sb_TF	Sb_Vis	Sbass_He	Sbass_TF	Sbass_Vis	TSB
X_m	2.02±0.20	1.77±0.16	1.85±0.18	1.96±0.14	1.65±0.18	1.78±0.26	1.69±0.20	1.93±0.14	1.79±0.24	1.89±0.12	1.76±0.15	1.68±0.18	1.85±0.15	1.72±0.13	1.71±0.16	2.40±0.23
v_m	0.24±0.09	0.15±0.08	0.16±0.07	0.24±0.07	0.18±0.10	0.13±0.05	0.12±0.04	0.13±0.03	0.11±0.05	0.21±0.08	0.16±0.07	0.09±0.07	0.12±0.06	0.11±0.04	0.08±0.06	0.20±0.09
λ_X	2.68(NS)	1.68(NS)	0.94(NS)	2.17(NS)	0.77(NS)	0.79(NS)	1.97(NS)	0.82(NS)	1.43(NS)	2.18(NS)	1.42(NS)	-0.03(NS)	0.73(NS)	1.41(NS)	0.78(NS)	1.01(NS)
μ_X	0.48±0.24	0.33±0.20	0.35±0.14	0.48±0.18	0.43±0.21	0.30±0.12	0.28±0.10	0.27±0.12	0.35(NS)	0.44±0.13	0.35±0.12	0.21(NS)	0.27(NS)	0.25(NS)	0.19(NS)	0.32±0.17
τ_X	6.88±1.45	7.71±1.76	6.60±1.62	6.36±1.98	5.41±1.87	7.55±2.01	9.05±1.90	8.32±1.63	9.37±2.32	6.69±1.54	7.06±1.32	9.68±1.65	8.22±1.79	9.33±1.55	11.1±2.4	7.18±0.99
t_{mX}	11.1±2.4	13.7±1.9	12.3±2.8	10.5±2.6	10.0±3.2	14.3±2.9	16.1±2.5	15.8±1.7	17.3±3.0	11.2±1.7	12.7±2.8	19.4±3.6	15.7±3.2	17.3±3.2	21.4±4.6	13.3±2.3
R^2	0.995	0.991	0.982	0.990	0.979	0.988	0.990	0.979	0.973	0.988	0.986	0.975	0.986	0.990	0.980	0.979
p-value	<0.0001	<0.0001	<0.001	<0.0001	<0.001	<0.0001	<0.0001	<0.0001	<0.0001	<0.0001	<0.0001	<0.0001	<0.0001	<0.0001	<0.0001	<0.0001
$Y_{X/RS}$	0.882	0.760	0.822	0.860	0.632	0.742	0.679	0.895	0.791	0.872	0.746	0.683	0.953	0.742	0.701	0.953
$Y_{X/Pr}$	1.09	0.842	0.931	1.12	0.751	0.792	0.732	1.08	0.860	1.08	0.904	0.783	0.997	0.841	0.771	1.30

Cells (G)

	Sa_He	Sa_TF	Sa_Vis	RT_He	RT_TF	RT_Vis	Tu_He	Tu_TF	Tu_Vis	Sb_He	Sb_TF	Sb_Vis	Sbass_He	Sbass_TF	Sbass_Vis	TSB
G_m	7.17±1.16	7.28±0.69	7.22±0.68	7.19±0.76	7.27±0.98	7.15±2.32	7.28±1.48	7.17±1.92	7.39±2.47	7.27±0.80	7.44±1.12	7.32±0.79	7.40±0.88	6.94±0.99	7.09±0.74	7.77±0.34
v_G	0.65±0.30	0.94±0.69	0.62±0.31	0.61±0.27	0.90±0.34	0.48±0.51	0.48±0.22	0.72±0.33	0.50±0.32	0.69±0.35	0.53±0.29	0.62±0.27	0.74±0.41	0.55±0.31	0.72±0.35	0.94±0.54
λ_G	1.36(NS)	2.36(NS)	2.17(NS)	1.35(NS)	2.20(NS)	1.85(NS)	1.19(NS)	1.88(NS)	-0.23(NS)	1.85(NS)	0.81(NS)	1.93(NS)	1.92(NS)	1.34(NS)	2.44(NS)	2.71(NS)
μ_G	0.36±0.15	0.52±0.21	0.35±0.14	0.34±0.20	0.50±0.23	0.27±0.22	0.26±0.16	0.40±0.18	0.27(NS)	0.38±0.21	0.29±0.18	0.34±0.16	0.40±0.24	0.32±0.20	0.40±0.21	0.48±0.29
τ_G	6.85±1.76	6.15±1.49	7.96±1.25	7.30±1.58	6.23±2.81	9.37±2.45	8.85±2.40	6.87±3.09	7.19±5.06	7.09±1.62	7.81±2.42	7.87±1.63	6.95±1.74	7.64±2.22	7.39±1.50	6.35±1.45
t_{mG}	12.4±3.8	10.0±3.9	13.7±5.4	13.2±5.7	10.3±4.2	16.9±4.6	16.5±4.9	11.9±5.8	14.6±8.3	12.3±3.6	14.8±5.6	13.8±3.7	12.0±3.8	13.9±5.1	12.3±3.3	10.5±3.1
R^2	0.947	0.989	0.982	0.974	0.973	0.985	0.985	0.982	0.957	0.969	0.955	0.975	0.964	0.958	0.974	0.966
p-value	<0.001	<0.0001	<0.0001	<0.0001	<0.001	<0.0001	<0.0001	<0.0001	<0.005	<0.001	<0.001	<0.005	<0.005	<0.001	<0.0001	<0.001
$Y_{G/RS}$	3.99	4.12	4.03	3.78	4.08	3.79	4.20	4.00	4.22	4.04	4.34	4.18	4.25	3.60	3.85	4.62
$Y_{X/Pr}$	2.70	3.02	2.98	2.80	3.09	2.84	3.24	2.94	3.19	3.16	3.25	3.13	3.02	2.74	3.01	3.36

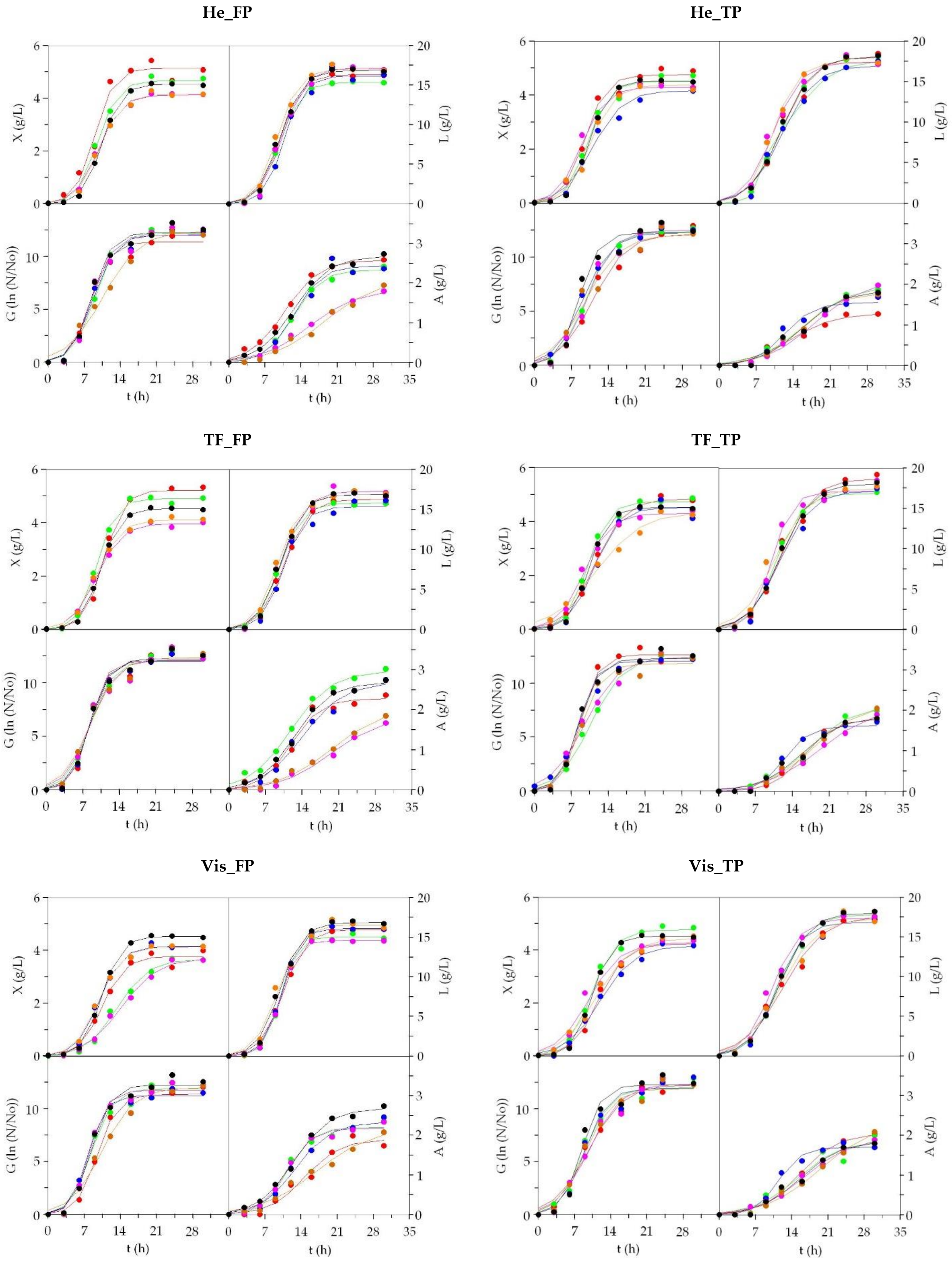

Figure S1. Fermentations of *L. brevis* in low-cost media based on peptones from aquaculture by-products. ●: salmon; ●: trout; ●: turbot; ●: seabream; ●: seabass; ●: MRS. Experimental data of biomass (X) and viable cells (G) were fitted to the logistic equation. The confidence intervals of experimental data (for two replicates) were in all cases less than 20% of the experimental mean values and omitted for clarity.

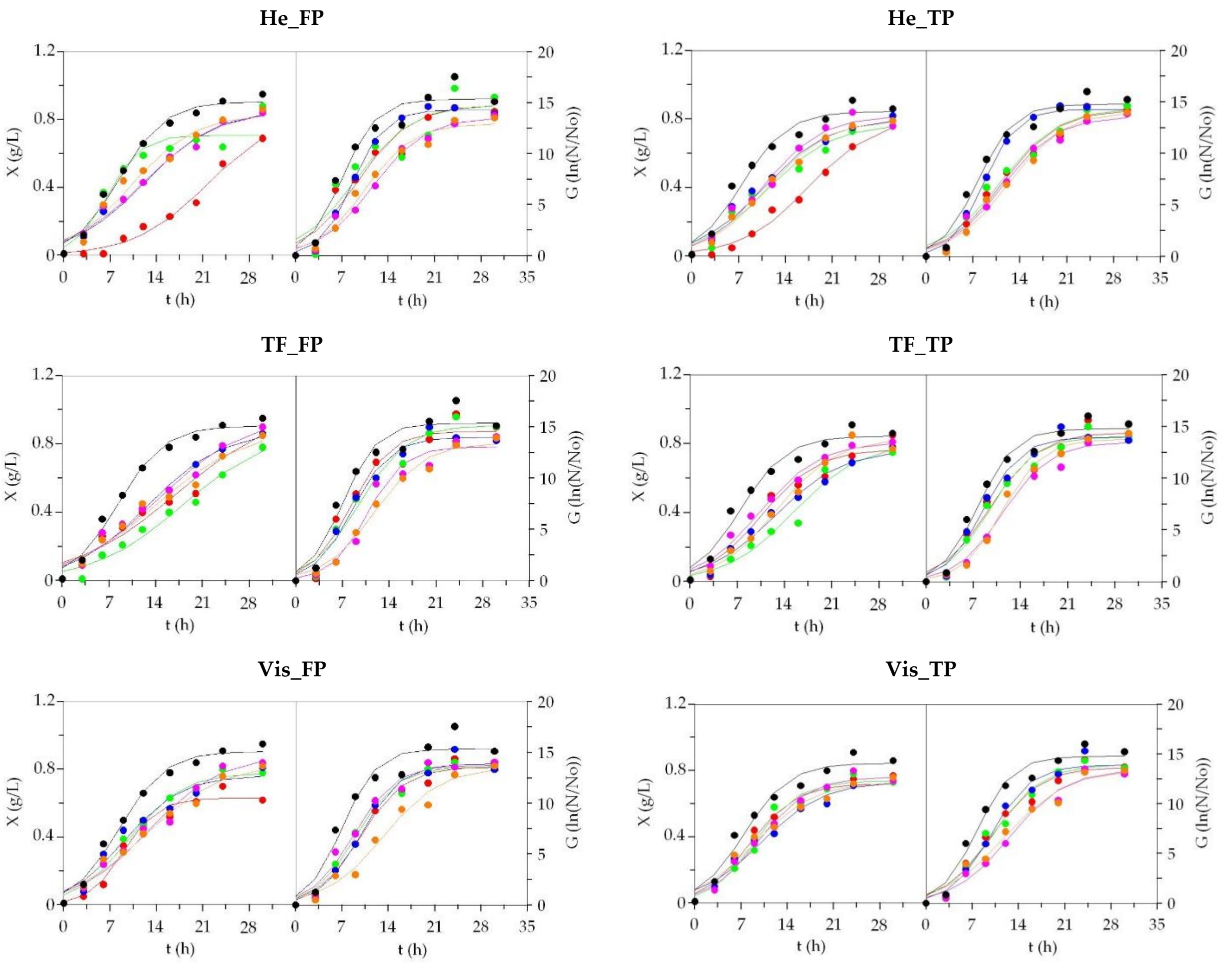

Figure S2. Growths of *P. fluorescens* in low-cost media based on peptones from aquaculture by-products. ●: salmon; ●: trout; ●: turbot; ●: seabream; ●: seabass; ●: MRS. Experimental data of biomass (X) and viable cells (G) were fitted to the logistic equation. The confidence intervals of experimental data (for two replicates) were in all cases less than 20% of the experimental mean values and omitted for clarity.

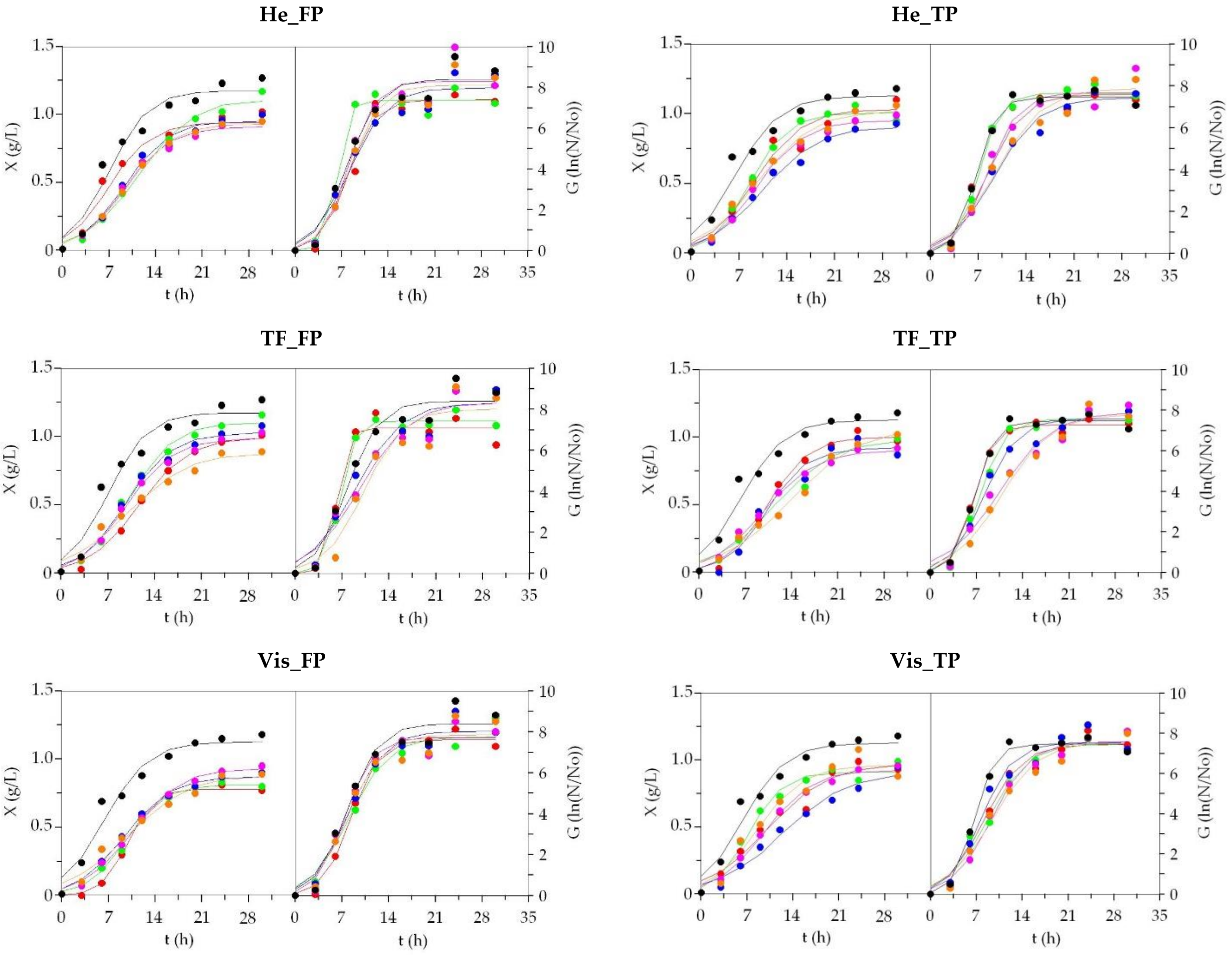

Figure S3. Growhts of *B. subtilis* in low-cost media based on peptones from aquaculture by-products. ●: salmon; ●: trout; ●: turbot; ●: seabream; ●: seabass; ●: MRS. Experimental data of biomass (X) and viable cells (G) were fitted to the logistic equation. The confidence intervals of experimental data (for two replicates) were in all cases less than 20% of the experimental mean values and omitted for clarity.

L. plantarum

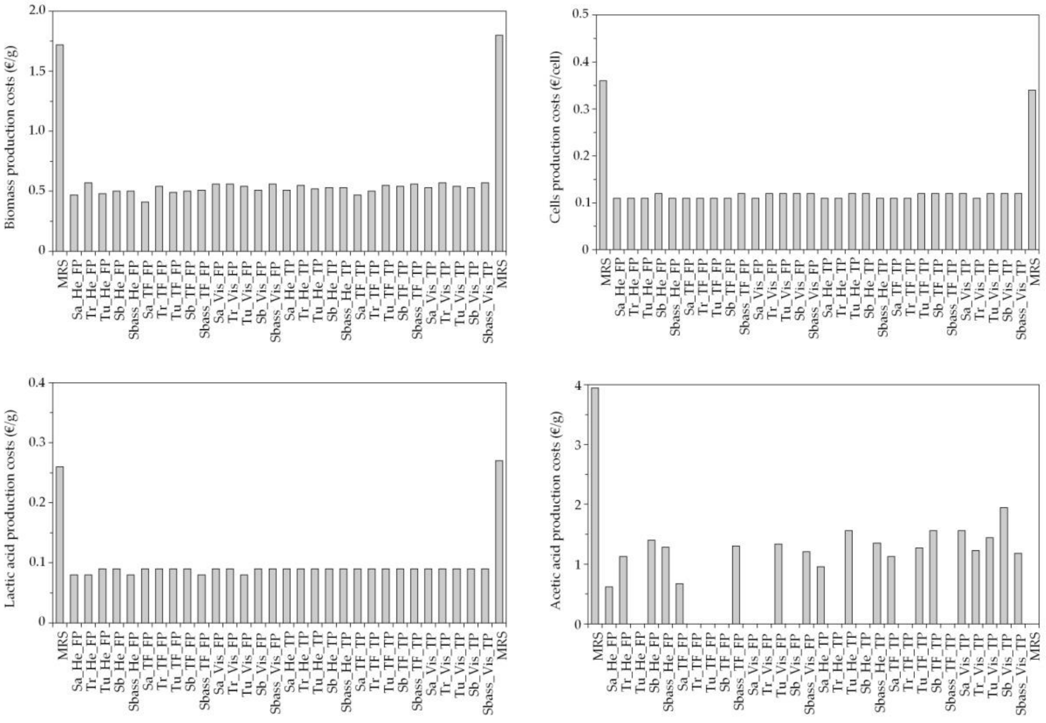

Figure S4. Economical evaluation of *L. plantarum* bioproduction costs in the nutritive media studied.

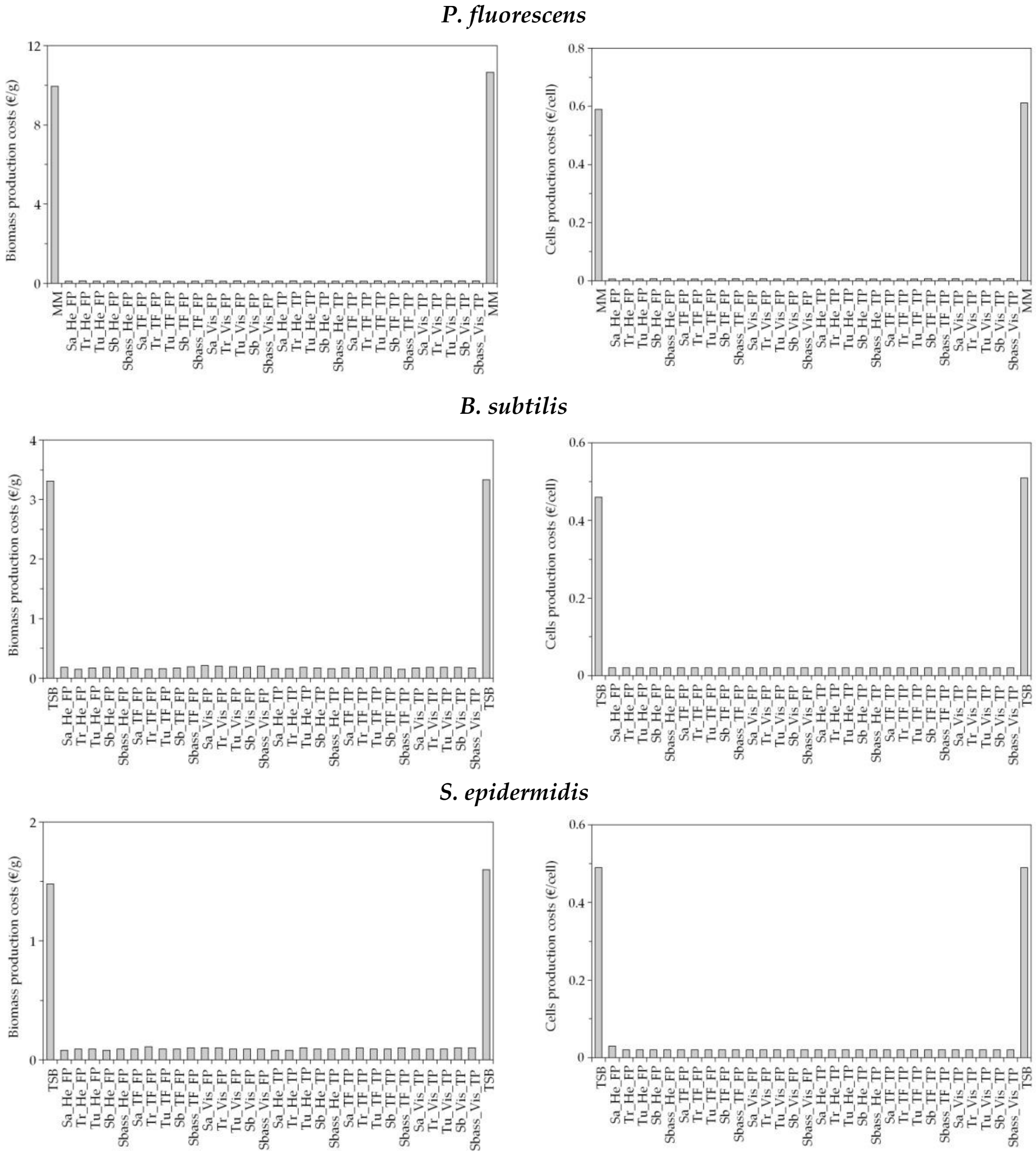

Figure S5. Economical evaluation of *P. fluorescens*, *B. subtilis* and *S. epidermidis* growhts costs in the nutritive media studied.
